# Recent Progress of Electrospun Nanofiber-Based Composite Materials for Monitoring Physical, Physiological, and Body Fluid Signals

**DOI:** 10.1007/s40820-025-01804-2

**Published:** 2025-06-18

**Authors:** Fang Guo, Zheng Ren, Shanchi Wang, Yu Xie, Jialin Pan, Jianying Huang, Tianxue Zhu, Si Cheng, Yuekun Lai

**Affiliations:** 1 College of Textile and Clothing Engineering, National Engineering Laboratory for Modern Silk, Soochow University, Suzhou, 215123 People’s Republic of China; 2 College of Chemical Engineering, Fuzhou University, Fuzhou, 350116 People’s Republic of China; 3 School of Materials and Chemistry, Anhui Agricultural University, Hefei, 230036 People’s Republic of China

**Keywords:** Flexible sensor, Electrospinning, Nanofibrous membrane, Composite materials

## Abstract

Flexible electronic skin (E-skin) sensors offer innovative solutions for detecting human body signals, enabling human–machine interactions and advancing the development of intelligent robotics. Electrospun nanofibers are particularly well-suited for E-skin applications due to their exceptional mechanical properties, tunable breathability, and lightweight nature. Nanofiber-based composite materials consist of three-dimensional structures that integrate one-dimensional polymer nanofibers with other functional materials, enabling efficient signal conversion and positioning them as an ideal platform for next-generation intelligent electronics. Here, this review begins with an overview of electrospinning technology, including far-field electrospinning, near-field electrospinning, and melt electrospinning. It also discusses the diverse morphologies of electrospun nanofibers, such as core–shell, porous, hollow, bead, Janus, and ribbon structure, as well as strategies for incorporating functional materials to enhance nanofiber performance. Following this, the article provides a detailed introduction to electrospun nanofiber-based composite materials (i.e., nanofiber/hydrogel, nanofiber/aerogel, nanofiber/metal), emphasizing their recent advancements in monitoring physical, physiological, body fluid, and multi-signal in human signal detection. Meanwhile, the review explores the development of multimodal sensors capable of responding to diverse stimuli, focusing on innovative strategies for decoupling multiple signals and their state-of-the-art advancements. Finally, current challenges are analyzed, while future prospects for electrospun nanofiber-based composite sensors are outlined. This review aims to advance the design and application of next-generation flexible electronics, fostering breakthroughs in multifunctional sensing and health monitoring technologies.

## Highlights

- This work reviews recent advancements in electrospun nanofiber-based composite materials for monitoring physical, physiological, and body fluid signals, with a particular focus on the design strategies of nanofiber-based composites.
- The electrospinning technologies, nanofiber morphologies, fabrication of nanofiber membranes, and the integration of nanofibers with materials such as hydrogels, aerogels, or metals are comprehensively reviewed and discussed.
- The current challenges and future prospects of nanofiber-based composite materials for human monitoring are discussed and analyzed.

## Graphical abstract

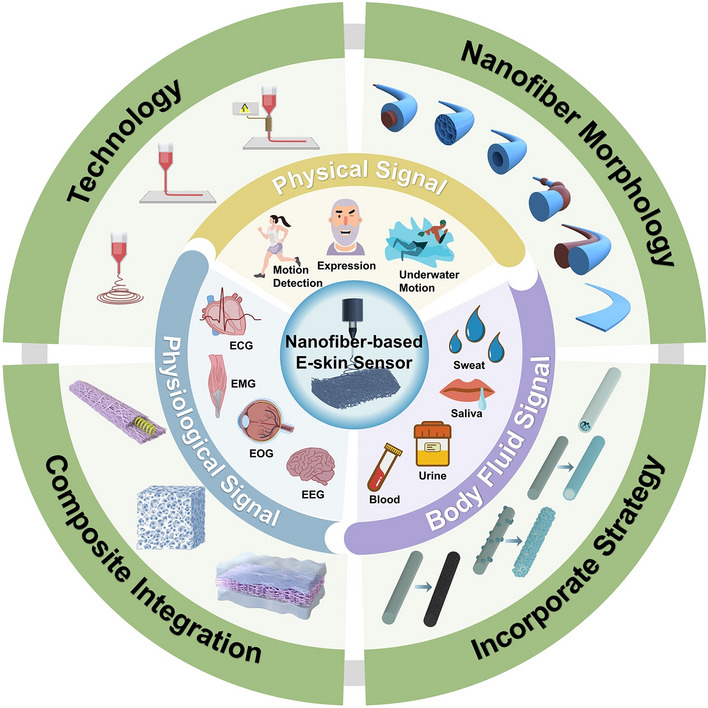

## Introduction

The human skin, the body’s largest organ, is crucial for sensing various external stimuli, including physical and chemical signals, and then transmitting these signals to the brain via nerves, enabling humans to respond and react to their environment [1–3]. In recent years, E-skin devices, which emerged as wearable technology that mimics the functionality of human skin while incorporating additional features, have attracted significant attention and research interest [4]. Currently, E-skin sensors are used in two main platforms: human body and intelligent robotics (artificial prosthetics). For human applications, sensors primarily detect various human signals including physical signals (i.e., pulse, joint movement, and facial motion), physiological signals (i.e., electrocardiograms (ECGs), electromyograms (EMGs), electrooculograms (EOGs), electroencephalograms (EEGs)), and body fluid signals (i.e., sweat, saliva, urine and blood) [5–7].

**Fig. 1:**
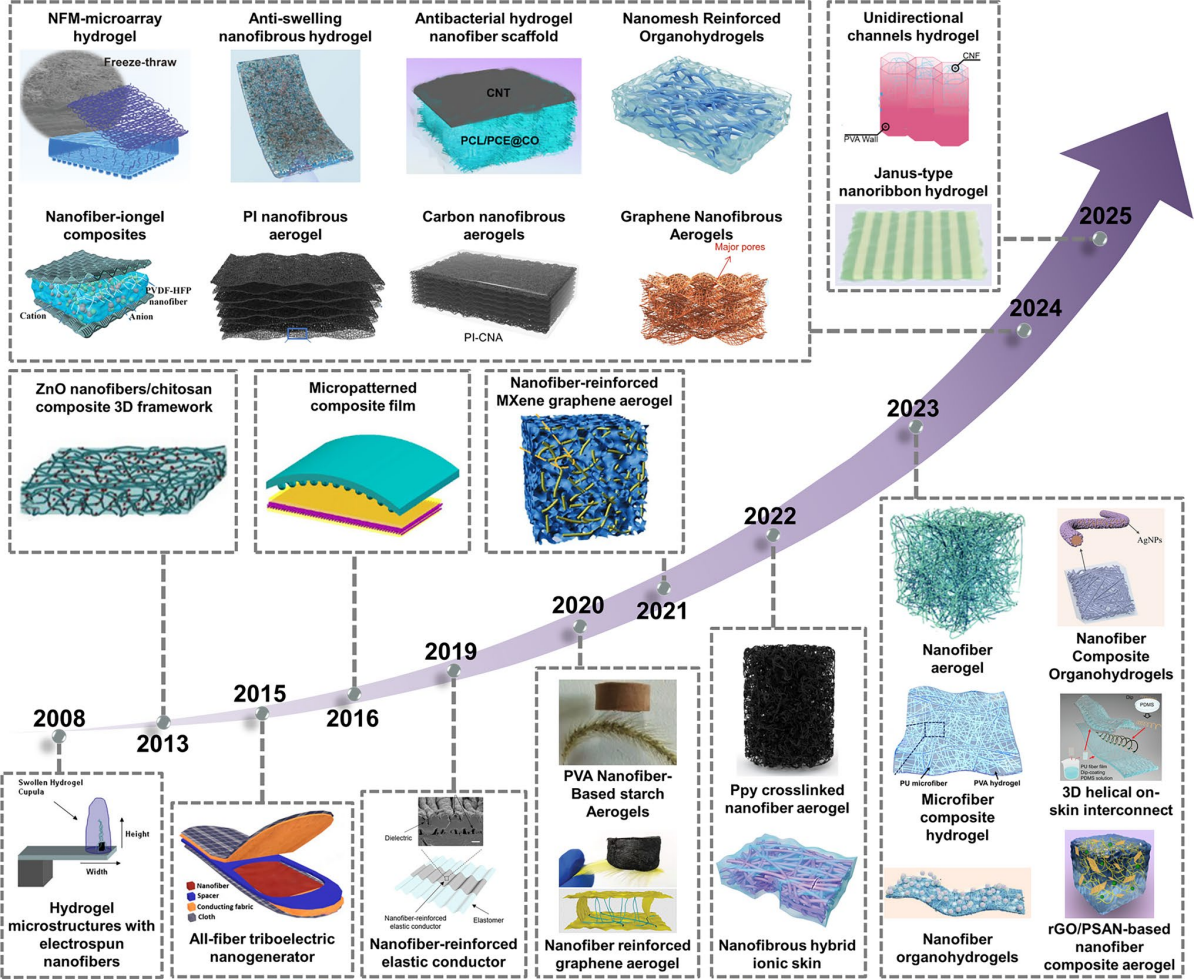
Summary of key historical developments of electrospun nanofiber-based composite materials in the field of flexible sensors at various states from 2000 to the present; Reproduced with permission from Refs. [8, 10–34]: Copyright 2008, Elsevier Ltd. Copyright 2013, Elsevier Ltd. Copyright 2015, Elsevier Ltd. Copyright 2016, Springer Nature. Copyright 2019, American Chemical Society. Copyright 2020, WILEY–VCH. Copyright 2020, WILEY–VCH. Copyright 2021, WILEY–VCH. Copyright 2021, Elsevier Ltd. Copyright 2022, The Author(s). Copyright 2023, Elsevier Ltd. Copyright 2023, The Author(s). Copyright 2023, The Author(s). Copyright 2023, WILEY–VCH. Copyright 2023, The Royal Society of Chemistry. Copyright 2023, WILEY–VCH. Copyright 2024, Elsevier Ltd. Copyright 2024, Elsevier Ltd. Copyright 2024, WILEY–VCH. Copyright 2024, American Chemical Society. Copyright 2024, American Chemical Society. Copyright 2024, WILEY–VCH. Copyright 2024, The Royal Society of Chemistry. Copyright 2024, Elsevier Ltd. Copyright 2025, Elsevier Ltd. Copyright 2025, American Chemical Society

Various types of materials have been developed and utilized for human sensing, among which composites of nanofibrous membranes (NFMs) with other materials have attracted widespread interest from researchers. Hydrogels, aerogels, and metal materials are often employed as composite components for electrospun NFMs. As the most commonly used form of E-skin sensors, hydrogels have inherent properties of high-water content that are similar to those of human tissue and variable mechanical properties. Integrating hydrogel with NFMs possesses the functional properties of hydrogel and the structural advantages of nanofibers (excellent mechanical properties and topological structures) [8]. In addition, the incorporation of NFMs significantly strengthens aerogels by creating a reinforcing network within the aerogel’s structure. This enhancement not only significantly improves the mechanical strength and durability of the nanofiber/aerogel composite materials, but also broadens their potential application in the field of flexible electronics [9, 10]. Furthermore, the integration of metal materials into NFMs enhances their compatibility with flexible electronics by enabling precise patterning and embedding of metal components within the NFMs structure. This facilitates the development of complex, multifunctional devices that can meet specific performance requirements and withstand challenging environmental conditions [11]. Since 2000, electrospun nanofiber-based composite materials have received extensive attention in flexible sensors, and different types of composite materials have been developed and applied to flexible devices, as illustrated in Fig. 1 [8, 10–34]. In early 2008, Kyle et al. introduced a composite of hydrogel microstructures integrated with electrospun nanofibers to enhance the sensitivity of flow sensing. Between 2008 and 2019, researchers explored straightforward composites combining nanofibers with fabrics, films, and rubber to create materials for monitoring human movement signals. From 2020 onward, electrospun nanofibers have been utilized both as reinforcements to improve toughness and as conductive components to enhance the electrical conductivity of composite materials. During this period, advancements in nanofiber-based composite materials have primarily centered around more greater assembly possibilities. Since 2023, the development of nanofiber-based composite materials for flexible sensing has increased significantly, and researchers have begun to explore additional possibilities for nanofibers, including anti-swelling effects, superior directional channels, and the rapid progress continues.

**Fig. 2:**
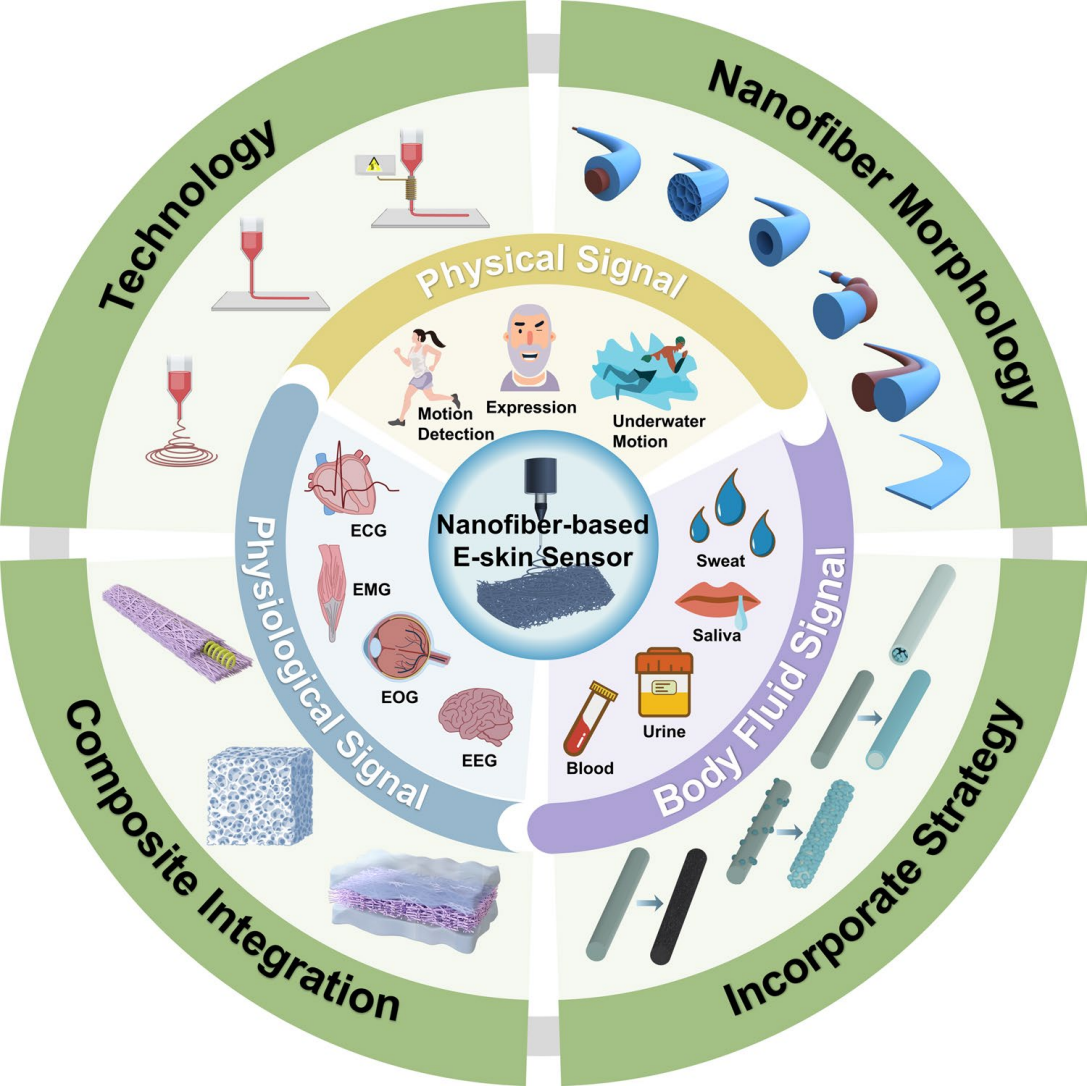
Schematic illustration of the aspects discussed in this review

Considering the rapid development of nanofiber-based composite materials for human signal monitoring applications, it is essential to make a comprehensive summary of this emerging field. This review aims to summarize recent advancements in electrospun nanofiber-based flexible sensors from three key aspects (Fig. 2). First, we discuss the latest progress in electrospinning technologies, including far-field electrospinning, near-field electrospinning, and melt electrospinning. The tuning of nanofiber morphology (i.e., core–shell, porous, hollow, bead, Janus, and ribbon structures) and the preparation of conductive nanofibers (i.e., mixing, coating, in situ growth, in situ polymerization, and carbonization) are also addressed. Subsequently, we explored the combination of electrospun nanofibers with other materials, such as hydrogels, aerogels, and metals. Finally, we highlight recent advancements in nanofiber-based composite materials for detecting various signals, including physical, physiological, and body fluid signals. This review thoroughly addresses the primary challenges facing nanofiber-based composite materials and provides critical insights into future development trends in this field.

## Electrospinning Technology and Electrospun Nanofibers

### Electrospinning Technology

Electrospinning is a straightforward, controllable, cost-effective, and scalable nanotechnology for producing nanofibers from natural and synthetic polymers [35–37]. Over recent decades of continuous innovation, three kinds of electrospinning technologies, far-field electrospinning (FFES) (conventional electrospinning), near-field electrospinning (NFES), and melt electrospinning (MES) have been developed. Table 1 summarizes the advantages and disadvantages of the different electrospinning techniques and the corresponding characteristics and application scenarios, which provides great guidance to fully understand the differences among the electrospinning technologies. Among these technologies, the FFES is the most widely used for producing large quantities of polymer nanofibers [38], which draw continuous fibers from a polymer solution through electrostatic force, forming a liquid jet. As this technique has been thoroughly discussed in numerous reviews, it will not be repeated in this paper [39–41].

**Table 1:** Summary of three electrospinning technologies and their characteristics

	Far-field electrospinning	Near-field electrospinning	Melt electrospinning
Technologies			
Principle	Spraying and stretching polymer solutions via high-voltage static forces	Reducing the nozzle-collector distance to reduce jet instability	Heating polymer to a molten state and stretching it into fibers within an electric field
Material compatibility	Compatible with various polymers	Compatible with various polymers	Compatible with thermoplastics materials
Fiber diameter/μm	Tens of nanometers to micrometers	Tens of nanometers to hundreds of micrometers	Micrometers to hundreds of micrometers
Safety	Risk of high voltage	Risk of electric field breakdown	Risk of high temperature
Advantages	High uniformity; Low cost; Easy process; Easy surface functionalization	Precise and controllable deposition; Ability to construct 3D structures; Large-area fiber patterns	Environment friendly; Solvent free; Controllable deposition; High yield; Prospects for industrial applications
Disadvantages	Difficult with direct writing; Consumption of organic solvent	Complex process; Low production speed; Consumption of organic solvent	High equipment complexity; High energy consumption; Degradation of heat-sensitive materials
Applications	Motion monitoring; Physiological monitoring; Health monitoring; Human–Computer Interaction; Biomedical diagnostics	Motion monitoring; Physiological monitoring; Health monitoring; Biomedical diagnostics	Physiological monitoring

NFES is a controllable technology for continuous ultrathin fiber production that has been developed in recent years [42–44]. In NFES, the distance between the needle and the collector is kept within a stable region, eliminating the whipped area of the liquid column and ensuring the jet remains straight [45, 46]. This technique allows for the production of neatly aligned, straight polymer fibers, with the bottom collector moving along a programmed path to create the desired pattern [47–49]. The unique advantages, like low applied voltage, high precision, and a short distance between the electrode and collector, enable the controllability over fiber deposition and the programmable manipulation of product features such as microstructure, thickness, and width [50–52]. Huang et al. [51] designed a polyurethane (PU) grid-like sensor with controllable thickness by NFES, which is sensitive only to the strain along the electrode direction but not to the perpendicular strain. This sensor can accurately detect hand movements and physiological signals. Additionally, NFES provides a unique method to prepare grid-like spacer layer structures for sensors which can improve the sensitivity and linear responsiveness of contact sensors by adjusting the period and thickness of the spacer layer [51].

MES technology is the process of extruding melted polymer in the presence of a strong electric field. The polymer material is usually thermoplastic, such as polypropylene (PP), polycaprolactone (PCL), polyethylene terephthalate (PET), or various resins [53]. Regularly patterned thinner fibers can be achieved by adjusting the melt flow rate, the melt temperature, and heating the surrounding air [54]. Melt electrowriting (MEW) is a specialized form of MES, where nanofibers are directed to the collector without a whipping effect [55]. Zhang et al. [56] self-made a novel three-dimensional mobile magnetic melt spinning device, replacing the electrostatic field with a magnetic field to eliminate safety concerns such as electric field breakdown. This device operates without an external high-voltage power supply, making it portable. While melt electrospinning has potential applications across various fields, its use in sensor technology has been relatively underexplored. Despite the numerous polymers available for FFES and NFES, MES is generally preferred for the production of polymer nanofibers from a production standpoint. Traditional FFES cannot accurately control the fiber layout but both MEW and NFES can produce materials with the ideal structure according to preset patterns.

### Microstructure of Electrospun Nanofibers

Researchers are increasingly focusing on the morphology and structural design of NFMs to achieve controllable signal transfer in E-skin sensors. Nanofiber with different microstructures can be obtained by adjusting electrospinning parameters, which in turn determine the mechanical and electrical properties of the sensors. Mechanical properties, surface roughness, and specific surface area of nanofibers with various microstructures are summarized in Table 2.

**Table 2:** Performance evaluation of NFMs with different microstructures

Nanofiber type	Components	Diameter/μm	Mechanical stress	Roughness	Surface area/m^2^/g	Refs
Core–shell structure	TPU/PVDF	0.6	Strain: 245% Stress: 13.5 MPa	Smooth	–	[57]
	PVP@TiO_2_	0.3–1	Strain: 2.5% Stress: 13 MPa	Smooth	–	[58]
	FLU-TPU/BFC-PVP	0.5	–	Smooth	–	[59]
	PDMS/BT@PVDF	0.084 ± 0.017	–	Smooth	–	[60]
	PU@Fe_3_O_4_-PU@CNT/Ag NPs/PDMS	0.5	Strain: 230% Stress: 19.31 MPa	Roughness	–	[61]
	PLLA/Gly	0.35 ± 0.02	–	Smooth	–	[62]
	CA + TPU/PPy	0.6–0.7	–	Roughness	–	[63]
Porous structure	In_2_O_3_@ZnO	0.05–0.2	–	Roughness	49.3115	[64]
	P(VDF-TrFE)	1–1.5	–	Roughness	–	[65]
	Pd-decorated In_2_O_3_ embedded SnO_2_	0.02–0.1	–	Roughness	53.001–53.952	[66]
	P(VDF-TrFE)	0.7–1	Strain: 110% Stress: 65 MPa	Roughness	–	[67]
Hollow structure	PVDF	0.367–0.637	Strain: 56.9% Stress: 8 MPa	Roughness	–	[68]
	Cu-doped In_2_O_3_	0.15–0.18	–	Roughness	–	[69]
	p-CuO/n-ZnO	0.5–1	–	Smooth	–	[70]
Beaded structure	PVA-PEDOT: PSS/TPU-CNT@MXene	< 0.05	–	Roughness	–	[71]
	TPU/CB	–	Strain: 90% Stress: 1.25 MPa	Roughness	–	[72]
	PVDF	0.8–1	–	Smooth	–	[73]
Janus structure	In_2_O_3_-NiO	0.07	–	Smooth	–	[74]
	[PVP/PANI]//[PAN/BTO]	0.45	–	Smooth	–	[75]
	[DF/GE]//[CB/GE]	4–5	Strain: 87.49% Stress: 0.769 MPa	Smooth	–	[32]
Ribbon structure	In_2_O_3_-Co_3_O_4_	0.6–1	–	Roughness	–	[76]
	WO_3_/FeWO_4_	0.3–1	–	Roughness	32.42–57.43	[77]

**Fig. 3:**
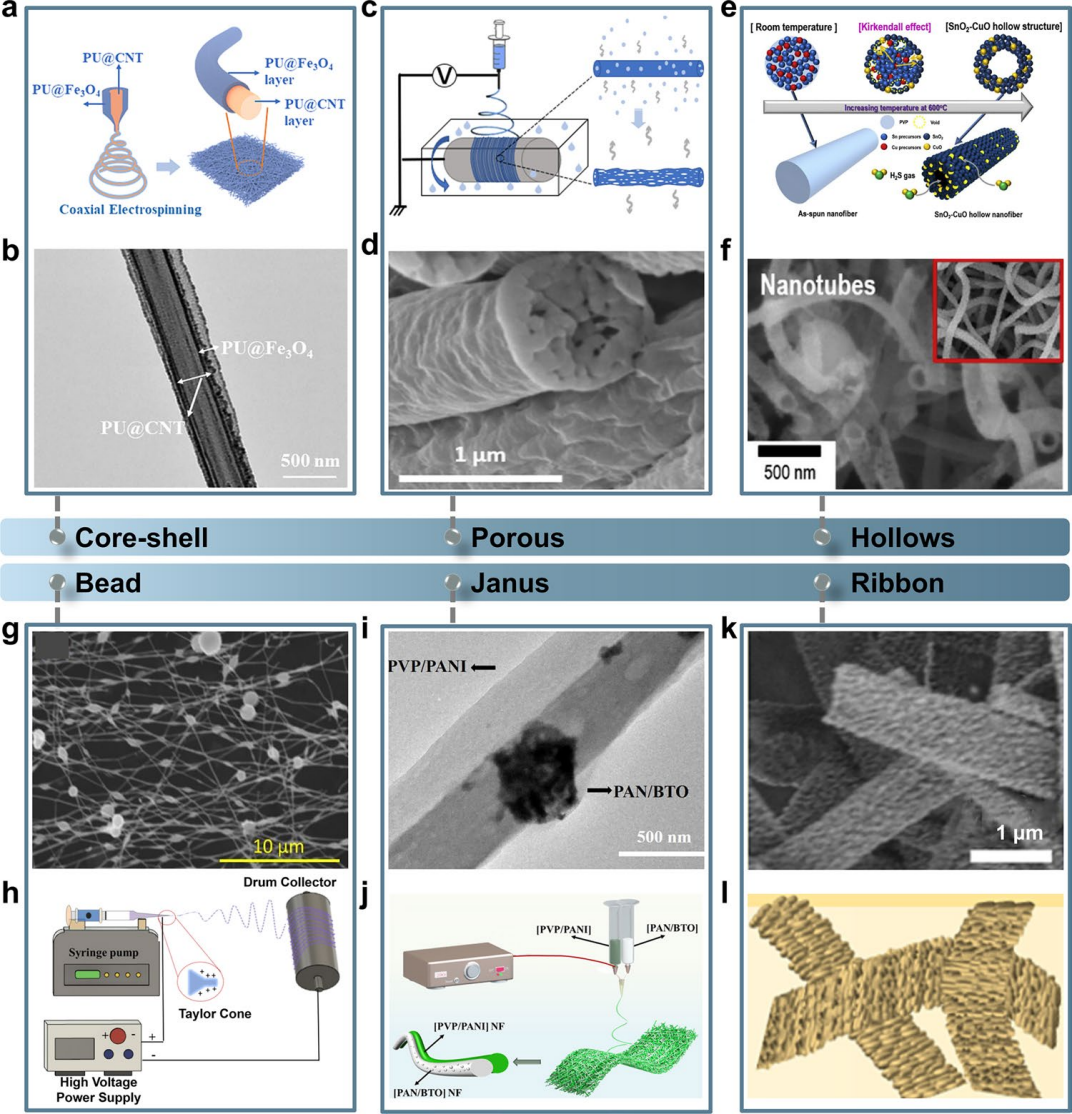
Schematic and SEM images of different types of electrospun nanofibers. **a, b** core–shell structure; Reproduced with permission from Ref. [61]: Copyright 2024, Elsevier B.V. **c, d** porous structure; Reproduced with permission from Ref. [67]: Copyright 2024, Elsevier Ltd. **e, f** hollow structure; Reproduced with permission from Ref. [86]: Copyright 2019, Elsevier B.V. **g, h** beaded structure; Reproduced with permission from Ref. [87]: Copyright 2024, American Chemical Society. **i, j** Janus structure; Reproduced with permission from Ref. [75]: Copyright 2022, Elsevier B.V. and **k, l** ribbon structure; Reproduced with permission from Ref. [77]: Copyright 2023, Elsevier B.V

*Core–shell structure* Nanofibers with a core–shell structure consist of two layers: an inner core and an outer sheath. This design not only enhances the mechanical strength of the nanofibers, but also introduces additional functionalities compared to single-layer nanofibers [59, 78–80]. Core–shell nanofibers are typically produced by a one-step coaxial electrospinning method, which simplifies the process by avoiding complex posttreatment [60, 81–84]. For instance, Fan et al. [61] developed core–shell NFMs using this method, where the CNTs in the core provide a continuous and stable conductive network, while the Fe_3_O_4_ in the shell offers abundant attachment sites for Ag NPs and imparts electromagnetic absorption properties. This device can effectively monitor fetal movements in pregnant women during the later stages of pregnancy (Fig. 3a, b). Core–shell nanofibers can endow unsinkable materials suitable for electrospinning by employing them as core materials, thereby facilitating the application of a broader range of materials [58]. Core–shell structured nanofibers can also form heterojunctions at the core–shell interface, enhancing gas sensing capabilities. Fu et al. [85] produced Co_3_O_4_/TiO_2_ core–shell nanofibers by coaxial electrospinning and annealing methods. The acetone gas sensing performance of core–shell NFMs was significantly improved attributed to the p-n heterojunction generated by Co_3_O_4_/TiO_2_.

Additionally, to form a core–shell structure, the decoration or deposition of functional materials on nonconducting nanofiber surfaces through polymerization has been widely explored [88]. This method preserves the flexibility of elastic polymers in the core layer, while allowing a conductive layer to be grown or deposited on the surface, thereby enhancing the electrical conductivity of the nanofibers [89]. The conductive nanofibers with a core–shell structure produced by this method can be utilized to create nanofiber-based aerogels. For instance, Chen et al. [63] prepared porous nanofiber aerogels (CA/TPU/PPy) by polymerizing PPy on nanofibers to create a rough conductive shell layer, followed by freeze-drying. The micro-bump structure of the conductive PPy shell not only enhances interfiber connections and prevents sliding and misalignment during compression, but also effectively concentrates the applied pressure on the sensitive tip area. Another innovative method for preparing core–shell structures is self-assembly [90]. For instance, Li et al. [62] facilitated the crystallization of poly(L-lactic acid) (PLLA) polymer chains using an interfacial anchoring strategy with PLLA/glycine (PLLA/Gly) nanofibers. This process primarily involved strong intermolecular interactions between the -OH groups on Gly and the C = O groups on PLLA, resulting in the formation of nanofibers with core–shell structures that displayed excellent piezoelectric properties.

*Porous structure* The introduction of pores in electrospun nanofibers can provide higher specific surface area, more active sites, enriched internal space and heterogeneous interfaces, which can accelerate the diffusion, transport or transformation of the detected substances [91]. Porous nanofiber structures can be obtained by two main strategies, namely template-assisted method and phase separation. The template-assisted method, which involves the electrospinning of sacrifice templates-contained precursor solution and template removal, is recognized as a simple and efficient approach for preparing porous nanofibers [92]. For example, Cai et al. [66] prepared SnO_2_ nanofibers into a porous structure with many small compression grains by electrospinning and thermal calcination. Based on the larger surface area due to the numerous porous, and the superabundance of connection points between grain boundaries, the SnO_2_ NFMs have good gas-sensitive properties. Additionally, high boiling point solvents such as DMSO [93, 94], mineral oil [44], and polymers like PEG [95] are beneficial for the formation of porous structures in nanofibers.

Vapor-induced phase separation (VIPS) is achieved by controlling humidity within the electrospinning device using humidifiers and dehumidifiers. In a high humidity environment, water vapor molecules condense on the surface of nanofibers, creating placeholders for the pores. When the water evaporates completely, a porous surface morphology is formed (Fig. 3c). The porous structure prepared by VIPS is an interior pores structure, while the surface forms a bark-like morphology due to the buckling instability of the polymer jet (Fig. 3d) [67]. Similarly, non-solvent induced phase separation (NIPS) is an important method for preparing porous nanofibers by electrospinning [96]. Lee et al. [65] utilized the difference in solubility parameters between the polymer and the solvent to facilitate the penetration of water molecules from the environment into the jet. This process induced phase separation of the homogeneous polymer jet. Subsequently, as the solvent evaporated during solidification, the polymer phase with embedded water molecules transformed into a porous structure, resulting in the formation of a Poly(vinylidene fluoride-co-trifluoroethylene)(P(VDF-TrFE)) porous nanofiber structure. The output voltage (85 V) of this porous nanofiber exceeds that of smooth surface fibers with high β-phase content. Furthermore, porous NFMs offer good benefits in acoustic power generation. The porous structure of NFMs has a lower acoustic impedance than ordinary nanofibers, allowing for increased sound absorption, decreased sound reflection, and greater conversion of sound energy into dynamic energy due to the presence of smaller pores. At the same time, the porous structure of the nanofibers has a lower modulus than a conventional smooth structure, resulting in stronger vibrations and deformations under the same sound stimulus, which produces a higher kinetic energy output and improves the sensitivity of acoustic power generation [95].

*Hollow structure* Nanofibers with hollow structure can be manufactured through coaxial electrospinning two different solutions, a coaxial spinneret with a soluble or volatile substance as the core layer and a polymer solution as the shell layer. Subsequently, the core component was then removed, leaving behind hollow nanofibers [68, 97]. The inner diameter of the nanofibers can be easily controlled by adjusting the solution concentration of the core solution. In Shao’s study [98], increasing the inner diameter of the polyvinylidene fluoride (PVDF) hollow nanofibers significantly enhances their volume deformation capability and consequently their piezoelectric output, achieving a piezoelectric output of 32.6 V for the hollow nanofibers, which is three times greater than that of the solid nanofibers. Another method to produce hollow nanofibers involves single-needle electrospinning followed by a calcination process [86, 99, 100]. The hollow-structured sensor material enhances the specific surface area and promotes the adsorption/desorption and diffusion of chemical molecules, showing great advantages in gas sensing [101]. For example, SnO_2_-CuO hollow nanofibers were fabricated by a combined process of electrospinning and calcination, which resulted in a uniform distribution of CuO nanoparticles (NPs) in SnO_2_ nanotubes [86]. The different diffusivities of the atoms in the diffusive coupling can lead to oversaturation of the lattice vacancies. As indicated in Fig. 3e, this oversaturation may lead to the condensation of additional vacancies near the interface in the form of “Kirkendall voids,” which in turn lead to the formation of nanotubes by calcination (Fig. 3f).

*Beaded structure* Beaded structure is typically achieved by reducing the concentration of the spinning solution. When the polymer solution concentration is low, spherical beads tend to form and connect with very fine nanofibers [71, 102]. However, the mechanical properties of these finer nanofibers are poor due to their beaded structure, rendering them unsuitable for application in strain sensor devices (Fig. 3g, h) [87]. As the concentration of the spinning solution is increased, the beads turn into spindle form, and the connecting nanofibers thicken due to greater attraction between the polymer chains. As the concentration of the spinning solution continues to increase, the beads disappear entirely, resulting in smooth nanofibers [103]. Another method to create a beaded structure is by mixing the microbead material with the spinning solution and then electrospinning the mixture. The porosity and compressibility of nanofiber membranes can be enhanced by incorporating large polymer beads into them. The microbeads were wrapped and anchored by the nanofibers, forming a porous structure that ensures the stable positioning of the microbeads within the membrane [73].

*Janus structure* The Janus structure generally involves injecting two different functional spinning solutions into two syringes in a one-step strategy, and then collecting the resulting Janus nanofibers with a collector. Compared with the core–shell structure, Janus nanofibers have two sides contacting with the environments that are useful for designing bifunctional sensors. For example, Hu et al. [75] reported an alternating current/direct current (AC/DC) dual-type signal device that combines the piezoelectric effect with dynamic Schottky contacts, addressing the compatibility of AC/DC outputs (Fig. 3i, j). This Janus nanofiber structure enables the collection and output of an AC voltage under a vertical force and a DC voltage signal under a horizontal slide. Additionally, Wang et al. [74] prepared Janus nanofibers through side-by-side electrospinning. This approach addresses the inhibitive effect of the shell layer on the core layer component in core–shell structures, allowing for a synergistic enhancement of the two materials’ properties. Moreover, the two components can react directly with the target molecules in the environment, leading to enhanced response. The Janus structure created through side-by-side electrospinning can also be utilized as a building block for hydrogels. Qi et al. [32] achieved the formation of highly aligned Janus nanofibers via high-speed roller collection, effectively segregating the conductive carbon black network from the fluorescent insulating materials. This aligned Janus structure enables the samples exhibit highly anisotropic electrical conductivity and pronounce fluorescent properties. The incorporation of Janus nanofibers enhances the anisotropy of hydrogel conductivity, exceeding that of conventional anisotropic conductive hydrogels. Additional studies have further demonstrated that the Janus structure improves the performance of anisotropic conducting hydrogels [104, 105].

*Ribbon structure* The ribbon structure can be achieved by adjusting the electrospinning parameters or adding functional materials. Generally, ribbon structures are formed when the spinning solution evaporates from the polymer solvent from the surface of the polymer fibers, leaving a solid film encapsulating the liquid core [106, 107]. Specifically, when the polymer precursor reaches the collector for a short time, a thin dry skin forms on the surface. Subsequently, the continuous evaporation of the solvent within the fibers causes the nascent round fibers to collapse, resulting in a banded cross section [76]. In addition, a ribbon structure can be formed by adding some functional materials. Wang et al. [77] transformed the morphology of the fibers from porous to widen hierarchical ribbons and the average diameter increased from 0.3 to 1 μm by continuously increasing the amount of MIL-101 added (Fig. 3k, l). A large number of WO_3_(001)/FeWO_4_(001) heterojunctions were distributed on the ribbon structure. This hierarchical configuration offers more active material loading sites, which can effectively enhance the sensitivity of the detected substances.

### Incorporation Strategies of Functional Materials in Electrospun Nanofiber

**Fig. 4:**
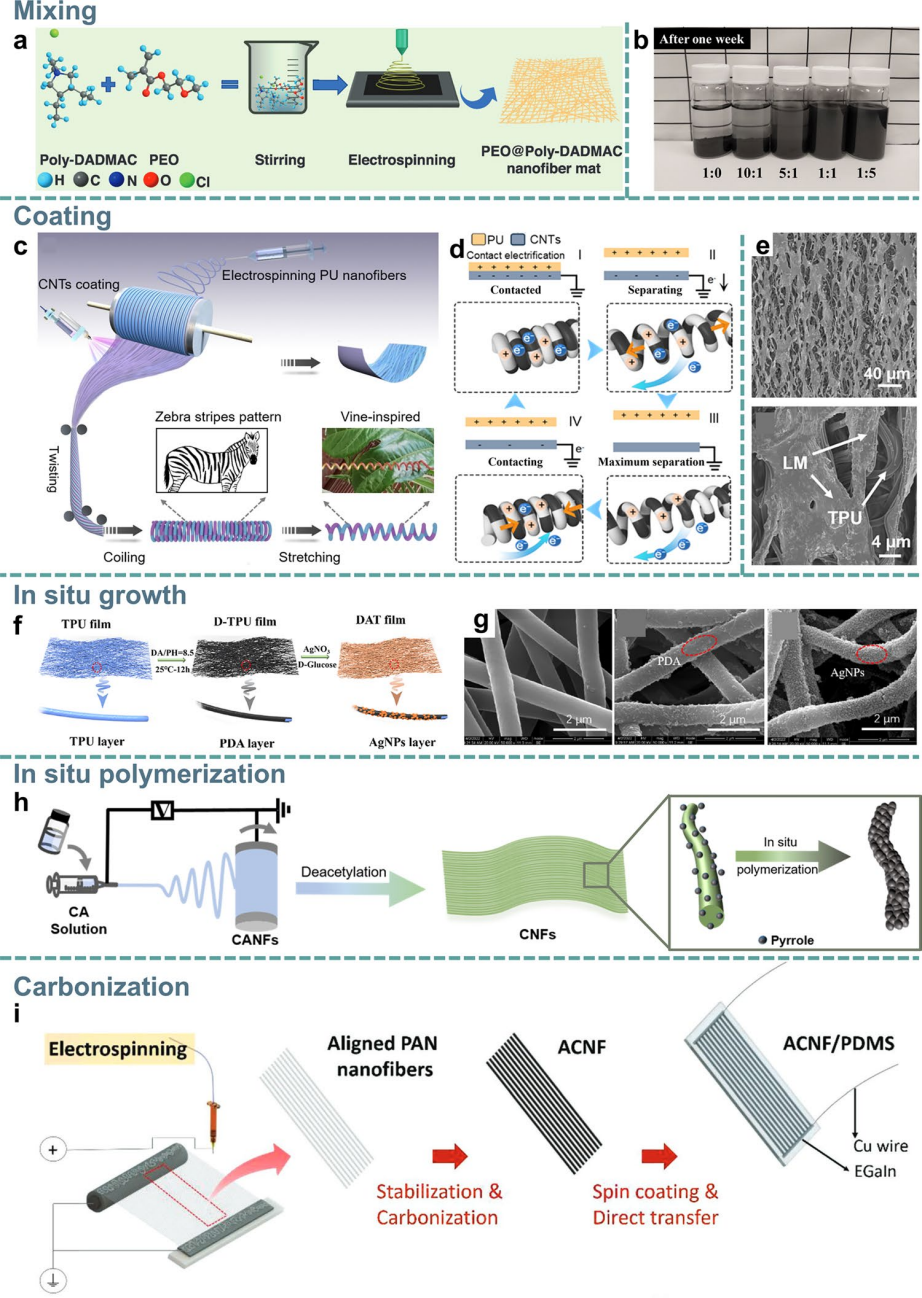
Fabrication methods of conductive electrospun nanofiber membrane. **a, b** Mixing method. **a** Schematic diagram of the process of the mixing preparation method; Reproduced with permission from Ref. [108]: Copyright 2024, Wiley–VCH. **b** Optical photographs of dispersion of rGO with Triton X-100 at weight ratios of 1:0, 10:1, 5:1, 1:1, and 1:5, respectively, after 7 days; Reproduced with permission from Ref. [109]: Copyright 2023, Springer Nature. **c-e** Coating method. **c** Vine-inspired structure design and manufacture process of the zebra-patterned TEHY. **d** Working mechanism of the single-electrode THEY; Reproduced with permission from Ref. [110]: Copyright 2024, American Chemical Society. **e** SEM images of LM on the aligned TPU NFMs; Reproduced with permission from Ref. [111]: Copyright 2024, Elsevier Ltd. **f, g** In situ growth method. **f** Schematic diagram of the process of the in situ growth method. **g** SEM images of the pristine TPU, D-TPU, and DAT NFMs; Reproduced with permission from Ref. [112]: Copyright 2024, American Chemical Society. **h** In situ polymerization method; Reproduced with permission from Ref. [113]: Copyright 2023, American Chemical Society. **i** Carbonization method; Reproduced with permission from Ref. [114]: Copyright 2019, WILEY–VCH

Electrospun NFMs can serve as functional components in nanofiber-based composite materials, including toughening components, sensing components, structural adjustment components, etc. To fabricate functional NFMs, researchers can incorporate functional nano/micro materials into polymers through various methods to develop functional NFMs for human signal monitoring. This section summarizes the strategies for incorporating functional materials into electrospun nanofibers: mixing, coating, in situ growth, in situ polymerization, and carbonization, as shown in Fig. 4.

#### Mixing

Nanofiber-based flexible sensors are often constructed by integrating conductive nano/micro fillers into soft elastomeric materials, creating percolation networks [115]. One effective method for producing conductive NFMs is mixing conductive fillers with polymer spinning solutions (Fig. 4a), which could avoid the detachment of conductive fillers from flexible nanofiber-based substrates effectively [108, 116–119]. The overall properties of flexible sensors are influenced by the size and the physicochemical characteristics of fillers and polymers [120–122]. A strong physical and chemical bond between the conductive fillers and polymer ensures a uniform and robust conductive network. Therefore, a comprehensive evaluation of the selection of conductive fillers and polymeric materials is necessary to make the optimal choice [123]. Conductive fillers are generally divided into zero-dimensional (0D, like metal NPs and carbon black (CB)), one-dimensional (1D, like carbon nanotube (CNT) and metal nanowires (NWs)), two-dimensional (2D, graphene and MXene), and amorphous fillers (conductive polymer, liquid metal (LM) and ionic liquid (IL)). In either state, it is required that the conductive fillers make contact with each other and provide a pathway for electrons to flow. Generally, 0D conductive fillers are sensitive to low deformation due to fewer contact points between the 0D conductive fillers. 1D conductivity fillers have high aspect ratios that facilitate the formation of the conductive pathways. 0D and 1D conductive fillers (metal and carbon-based) exhibit excellent electrical conductivity but have lower compatibility with polymer elastomers, which can directly affect the mechanical properties of NFMs. Li et al. [120] incorporated CNT fillers into PVDF, enhancing Young’s modulus while reducing the elongation of the nanofiber membranes. Due to its high negativity and excellent conductivity, the 2D MXene serves as an effective filler for enhancing the dielectric properties of polymer materials, thereby improving their triboelectric output performance [124]. However, the presence of hydroxyl groups on the MXene makes it susceptible to oxidation and subsequent loss of conductivity and usually requires antioxidant treatment or encapsulation to reduce oxidation [125]. Compared to MXene, graphene has more stable conductivity properties [126]. In addition to these fixed shape conductive fillers, amorphous IL and LM have also been widely studied for soft and flexible electrodes because of their extensibility, extremely high conformability, and low impact on the mechanical properties of NFMs [127–129].

On the other hand, obtaining the homogeneously distributed nanomaterials in the matrix is essential, as agglomeration resulting from inhomogeneous dispersion can significantly impact the mechanical performance of the membranes seriously. Typically, physical methods including ultrasonication and magnetic stirring are employed to create a uniform mixture [120, 130, 131]. However, some materials tend to agglomerate again after stopping stirring for a while. To address this issue, surface dispersants such as dimethyl sulfoxide (DMSO), sodium dodecyl sulfate (SDS), and Triton X-100 can be added to enhance the dispersion of these materials within the polymer matrix [132]. Li et al. [109] proposed self-healing silver NPs/reduced graphene oxide/3,4-polyethylene dioxythiophene:polystyrene sulfonate/TPU (Ag NPs/rGO/PEDOT:PSS/TPU) nanofiber electrodes for ECG and EMG monitoring. Triton X-100 promoted the dispersion of rGO, and Ag NPs in the PEDOT:PSS solution, resulting in enhanced electrical conductivity. By observing the dispersibility of different rGO:Triton X-100 ratios (1:0, 10:1, 5:1, 1:1, and 1:5), it was found that the ratios of 1:1 and 1:5 had better dispersibility without significant precipitation (Fig. 4b). Additionally, Li et al. [120] used SDS to disperse multi-walled CNT (MWCNTs) in a polymer solution, enhancing their distribution. Electrospun solutions with good dispersibility have improved both the electrospinning operation and the electrical conductivity and mechanical properties of NFMs.

#### Coating

Coating is a widely used and economical method for preparing nanofiber-based flexible sensors, allowing for surface modification of nanofiber membranes with few limitations on the choice of coating conductive materials. Various coating methods are available, including dip coating [133, 134], spray coating [135–137], ink trace and screen printing [138, 139], ultrasonication [140–142], sputtering [143–145], etc. Table 3 summarizes the advantages and disadvantages of the representative coating methods. Spray coating is highly versatile and can be used to create patterned structures through masks or to achieve Janus structures through single-sided spraying. Gao et al. [110] developed superelastic triboelectric helical yarn (TEHY) by unilaterally spraying CNTs dispersion on PU substrate and subsequently twisting them (Fig. 4c). A zebra pattern is formed between the black conductive CNT and the white insulating PU nanofibers, and a potential difference is generated between the CNT and the PU nanofibers during stretching and releasing, which can be used as a flexible sensor to monitor the human body movement (Fig. 4d). Another coating approach involves electrospinning the polymer into a liquid bath with conductive materials instead of a conventional solid collector, known as wet electrospinning, which can directly coat the polymer surface with conductive material [146]. These methods allow for the secure anchoring of nano-/micro-dimension coating materials on the rough surface of the electrospun NFMs layer [147]. The drawback of the coating method is that the conductive layer may affect the air permeability of the membrane to a different extent, which can directly influence the wearing comfort of the flexible sensor [148].

**Table 3:** Advantages and disadvantages of coating methods

Methods	Advantages	Disadvantages	Refs
Dip coating	Easy operation; High production efficiency; Uniform coating; Low cost	Uncontrollable thickness; Some substrates unsuitable for immersion	[133, 134, 149, 150]
Casting	Uniform coating	Defect-prone; Limited precision	[151]
Spray coating	Rapid; Uniform coating; Suitable for complex surfaces	Paint waste	[135–137, 152]
Spin coating	Highly controllable thickness; Uniform coating; High production efficiency	Defect-prone	[153, 154]
Screen printing	Rapid; Personalized preparation; Fast printing speed; Repeatable	High ink requirements; Poor printing precision; Ink waste	[138, 139]
Vacuum filtration	High efficiency	Unsuitable for viscous materials	[150, 155, 156]
Ultrasonication	Strong substrate bonding; Uniform coating; Wide applicability	Heat-susceptible	[140–142]
Sputtering	Ultrathin conductive layer; Shape preservation; Permeability	Low sputtering rate	[143–145]
Evaporation	High coating quality; Easy operation	Heat-susceptible; Material restrictions	[157]
Electrospray	High utilization; Fast droplet settling speed; Good particle dispersed	Poor penetration	[158, 159]
Pressure-stamp	Precision Circuitry; Personalized preparation; Rapid	Expensive raw materials	[160]

Through the coating method, the conductive materials may penetrate into the pores of the NFMs, forming the interpenetrating structure between the nanofibrous substrate and coating materials, which enhances the stability of conductivity (Fig. 4e) [111]. For instance, Yu et al. [161] developed a dynamic liquid metal-microfiber interlocking interface by depositing Cu particle-mixed eutectic gallium-indium liquid metal (Cu-EGaIn) onto nanofiber membranes through scrape-coating. The Cu-EGaIn is semi-embedded within the porous elastic microfiber membrane, and through a subsequent rolling-up process, metastructured nanofiber membranes with self-encapsulated conductive pathways are formed. The specially designed structure exhibited exceptional electrical conductivity (1.5 × 10^6^ S m^−1^) at the interface where the liquid metal interacts with the micro/nanofibers, particularly at their interlocking junctions. In addition, the conductive material can also be firmly bonded to the substrate material through chemical forces such as hydrogen bonding [162, 163]. A uniform and strong conductive layer is essential to ensuring the accuracy of human signal transmission. Apart from the physical embedding and hydrogen bonding of the conducting material with the substrate material, additional protective layers or adhesives around the conductive layer have been widely applied to enhance the strength of the conductive-substrate bond [110]. Polydimethylsiloxane (PDMS) is commonly used as an environmentally friendly encapsulation material to ensure uniformity of the device while providing protection against the ingress of sweat and other liquids into the sensing system and thus ensuring accurate test data [164]. Additionally, polydopamine (PDA) is particularly effective in improving interfacial adhesion, which is essential for achieving a stable and uniform conductive layer during the coating process [165, 166].

#### In Situ Growth

Conductive fillers will be in situ synthesized from metal ions on flexible substrates uniformly through chemical reactions during the in situ growth process [167]. In situ growth enables uniform deposition of conductive particles on the fiber surface with high reaction stability and high deposition efficiency. Wu et al. [112] reported a breathable nanofiber flexible sensor (named DAT) by dispersing the metal precursor solution on the surface of the PDA-modified TPU NFMs (D-TPU) followed by in situ growth uniform Ag NPs without agglomeration (Fig. 4f). This technology enables electrospun NFMs to maintain large pore size and high specific surface area, while also displaying high surface coating rates and dense outer conductive layers (Fig. 4g). Direct growth of Ag NPs on PVDF nanofibers presents challenges due to electrostatic repulsion and the rapid reduction of Ag^+^. To address this, Au seed-mediated synthesis can facilitate the reduction of Ag^+^, leading to the formation of Ag NPs on nanofibers with high curvature [167]. However, excessive precursor concentrations can lead to the agglomeration of in situ-grown particles, consequently reducing the homogeneity of the composite film. Zhu et al. [121] demonstrated that when the concentration of Zn(NO_3_)_2_⋅6H_2_O was too high, ZnO NPs tended to agglomerate, resulting in stress concentration during the stretching process. This agglomeration phenomenon adversely impacts the overall mechanical properties of the composite film, thereby affecting stress transfer during the piezoelectric process. Therefore, it is necessary to optimize the precursor concentration to enhance material properties. In addition, in situ growth significantly influences the thickness and the roughness of the nanofibers. The deposition of Co(NO_3_)_2_ on the surface of the polyacrylonitrile (PAN) nanofibers results in a complete encapsulation of the nanofiber surface with ZIF-67 NPs, leading to an increase in fiber diameter [168]. Simultaneously, the development of a rough surface structure through in situ growth improves the sensitivity of the flexible sensor.

#### In Situ Polymerization

In situ polymerization is a technique used to bond conductive polymers to the surface of NFMs, which serves as templates for the in situ polymerization of aniline, pyrrole, and EDOT monomers to obtain conductive polymers, to impart electrical conductivity to nanofiber membranes (Fig. 4h) [169–172]. The size of polymer particles is positively correlated with the concentration of the polymerization solution and the polymerization time. For example, Yan et al. [173] controlled the polymerization of PANI on PAN for varying durations, observing that the size of the polymerized particles gradually increased with longer reaction times, while the electrical conductivity of the fiber membrane was also enhanced. However, excessive concentration can lead to particle aggregation, reducing the uniformity of the composite membrane and diminishing its overall mechanical properties [174]. It is worth noting that high temperature accelerates the polymerization reaction, whereas low temperatures favor the formation of ordered structures, which reduces structural defects and enhances electrical conductivity [19]. Contributed to the stable reduction reaction, even distribution of conductive polymer particles, and high deposition efficiency, in situ polymerization is easier to scale up. In addition, the hydrophilicity of the fiber membrane surface can be effectively changed by in situ polymerization reaction [175]. For example, Li et al. [113] reported hydrophobic and rough PPy@cellulose nanofibers treated by in situ polymerization PPy. Due to the hydrophobicity of PPy as well as the rougher surface of the nanofibers after in situ polymerization, the contact angle of polymerized NFM was increased from 39° to 125.6°. This technology offers significant potential for functionalizing electrospun nanofibers, addressing the limitations of uneven distribution of functional materials on the surface of nanofiber membranes and preventing pore clogging.

#### Carbonization

Carbonized nanofibers (CNFs), obtained through high-temperature carbonization, are considered ideal candidates for sensor materials due to their large specific surface area, high porosity, and good electrical conductivity [176]. So far, PAN has been extensively utilized in carbonization technology due to its unique molecular structure characteristics. The molecular chains of PAN primarily consist of carbon elements with minimal branching. This composition maximizes the integrity of the three-dimensional conductive flexible network formed by carbon chains following carbonization [177, 178]. Cai et al. developed a flexible and highly sensitive pressure sensor using Fe_3_O_4_/PAN nanofibers through electrospinning and carbonization [179]. During the carbonization of PAN, graphitic layers develop, releasing gases like N_2_, HCN, and H_2_O [180]. Additionally, the temperature at which carbonization occurs significantly affects the mechanical and electrical properties of the nanofiber membranes. Lee et al. [114] reported a highly aligned, multidirectional and conductive anisotropic carbon nanofiber (ACNF) membrane by carbonizing aligned PAN NFMs (Fig. 4i). The electrical conductivity of the ACNF membrane increased with rising carbonization temperatures: from 0.0012 S m^−1^ at 600 °C to 5.72 S m^−1^ at 800 °C, and further to 125.25 S m^−1^ at 1000 °C. At temperatures above 1000 °C, the ACNF film, which is around 10 μm thick, became extremely brittle, making it difficult to embed into the PDMS substrate. However, the electrical conductivity of CNFs is significantly affected by the carbonization process, making precise control of conductivity challenging [176]. Moreover, the application of pure CNFs in flexible sensors is severely restricted due to the reduced flexibility of NFMs after carbonization. To enhance the flexibility of NFMs, researchers have explored various modifications to CNFs during or after the carbonization process. Liu et al. [181] improved the flexibility of CNFs by incorporating Bi_20_TiO_32_ (BT) into PAN. This enhancement is primarily attributed to the abundance of BT particles distributed throughout the fibers and their interiors, forming a dense interconnecting layer. After high-temperature carbonization, these particles function as a structural backbone, preventing significant shrinkage and deformation of the composite carbon/Bi_20_TiO_32_ (CBT) nanofibers. Simultaneously, BT reduces the brittleness of CNFs by effectively distributing stress. In addition to PAN, poly(vinyl alcohol) (PVA) and cellulose can also serve as carbon precursor polymers for the formation of nanofibers, enabling the synthesis of CNFs through a subsequent carbonization process [182, 183].

Table 4 summarizes the advantages and disadvantages of five incorporation strategies for conductive nanofibers. The choice of fabrication methods for nanofiber membranes significantly influences their performance characteristics, including conductivity, mechanical properties, and overall functionality. Future research should aim to optimize these methods and explore the use of combined approaches to enhance the performance of NFMs.

**Table 4:** Advantages and disadvantages of various incorporation methods for conductive NFMs

Methods	Advantages	Disadvantages
Mixing	Stable conductive filler; breathability; continuous nanofiber structure; high porosity	High homogeneity requirement in solutions; limited conductivity
Coating	Wide choice of conductive materials; multifarious methods; controllability; maintains substrate mechanical properties	Low breathability; weak adhesion between coating and substrate
In situ growth	Uniform deposition; strong bonding to substrate; breathability	Strict process conditions
In situ polymerization	Uniform distribution of conductive components; high adhesion to substrate	Difficult to control polymerization parameters; load amount is poorly controlled
Carbonization	Excellent conductivity; excellent thermal and chemical stability	Decreased flexibility; restricted precursor options

## Electrospun Nanofiber-Based Composite Materials

2D NFMs can be further transformed into 3D structures by integrating them with a variety of bulk materials. These materials include hydrogels, which provide a soft and flexible matrix, aerogels that offer a lightweight and porous framework, and metals, which can enhance the structural stability and conductivity of the composite.

### Nanofiber/Hydrogel Composite Materials

**Fig. 5:**
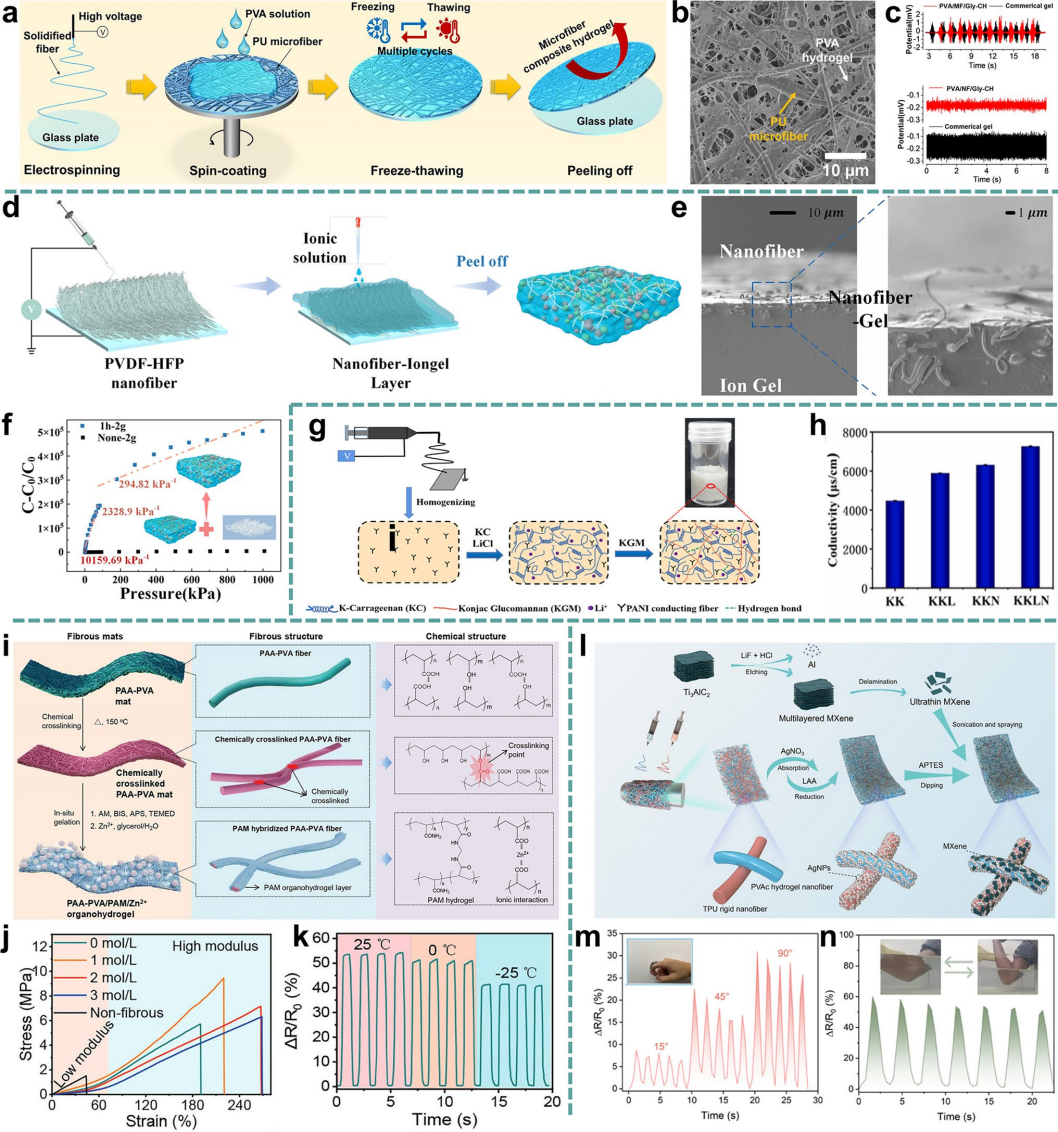
Nanofiber/hydrogel composite materials. **a** Preparation process of nanofiber-based hydrogel via spin coating and freeze-thawing.** b** SEM image of the composite hydrogel surface. **c** Performance comparison of EMG biosignals collected by the composite hydrogel and commercial gel; Reproduced with permission from Ref. [21]: Copyright 2023, Springer Nature. **d** Preparation of skin-inspired NFIG sensor by drop-casting method. **e** Surface micrograph of NFIG. **f** Capacitive response of the sensor at different pressures; Reproduced with permission from Ref. [25]: Copyright 2024, Elsevier Ltd. **g** Preparation process of nanofiber-based conductive hydrogel via direct filling and cooling method. **h** Conductivity of the different types of conductive hydrogel; Reproduced with permission from Ref. [186]: Copyright 2022, Elsevier B.V. **i** Construction of nanofibrous composite organohydrogels. **j** Stress–strain curves of the nanofibrous organohydrogels prepared under different AM concentrations and the nonfibrous organohydrogels. **k** Resistance response of the nanofibrous organohydrogels to cyclic strain of 80% at different temperatures; Reproduced with permission from Ref. [22]: Copyright 2023, Wiley–VCH. **l** Fabrication of TPAMH nanofibrous hydrogel composites. **m** Relative resistance changes are obtained from the motion of the finger. **n** Underwater detection of the elbow bending; Reproduced with permission from Ref. [30]: Copyright 2024, The Royal Society of Chemistry

Hydrogels are crosslinked polymer networks that contain a significant amount of water molecules with stretchability, fracture toughness, conductivity, and compatibility with biological tissues, making them ideal candidates for flexible bioelectronic devices [21, 28]. By combining hydrogels with electrospun nanofibers, the stability, recoverability, and durability of hydrogels can be effectively improved, and the combination property of the resulting nanofiber/hydrogels makes them promising for sensing applications [184, 185]. There are various methods to fabricate electrospun nanofiber/hydrogel composite materials, including the following: (1) Combining individually fabricated electrospun nanofibers and hydrogel by spin coating or drop coating hydrogel materials. Coating a thin hydrogel layer onto the surface of electrospun NFMs and then forming a tightly bound bilayer nanofiber/hydrogel composite materials by physical crosslinking or entanglement is a representative approach [24, 26, 28]. The interlocking structure between the NFMs and the hydrogel, along with strong hydrogen bonding, contributes to an effective combination between the two materials [26]. Gao et al. reported an ultrathin composite hydrogel with broadly tunable mechanical strength that matches most human living tissues [21]. The hydrogel composite material was composed of embedding PU microfibers into the PVA hydrogel through electrospinning, spin coating, and freeze-thawing methods (Fig. 5a, b). The interfacial linkage between the PU nanofibers and PVA was enhanced due to the formation of strong hydrogen bonding between the hydroxyl groups of PVA and the urethane group of the PU nanofibers. The incorporation of the NaCl and glycerol (Gly) impart the nanofiber-hydrogel composite ionic conductivity, anti-freeze, and anti-dehydration properties and the composite electrode exhibited lower background noise than a commercial gel electrode (Fig. 5c). Additionally, ionic gels exhibit similar modulus and ion migration characteristics with human skin. A nanofiber gel (NFIG) composite was developed by integrating high-modulus NFMs with an ionic gel matrix [25]. As illustrated in Fig. 5d, the ionic gel in drops was dipped onto high-modulus elastic NFMs made of PVDF-HFP using a dropwise maneuver. The NFIG surface features a nanofibrous texture mimicking human skin’s topography for enhanced sensitivity, while its internally embedded nanofiber gel matrix, analogous to dermal collagen, provides structural integrity against deformation. A soft ionic gel substrate enables robust pressure tolerance (Fig. 5e). The obtained NFIG exhibits an ultra-high sensitivity of 10159.69 kPa^−1^ and a wide pressure range of about 1000 kPa (Fig. 5f). (2) Broken up the NFMs with a high-speed homogenizer and added to the hydrogel solution to form a nanofiber/hydrogel. For example, Chen et al. [186] reported a biologic interpenetrating network hydrogel (KKLN) by introducing Li^+^ and electrospun polyaniline (PNAI) fiber (which served as conductive additives) in K-Carrageen (KC) and Konjac glucomannan (KGM) hydrogel matrix (Fig. 5g). PANI fibers were dispersed at high speed in a homogenizer are mixed with KGM/KC (KK) solution to form a stable hydrogel solution. The electrospun PANI nanofibers in the hydrogel accelerate electron transport to achieve higher electrical conductivity than KK hydrogel (Fig. 5h). This improvement is crucial for the rapid formation of the gel and for preventing the accumulation of LM droplets, ultimately providing superb conductivity. (3) In situ gelation hydrogel materials on the nanofibrous membrane surface. A new type of nanofiber-based organohydrogels poly(acrylic acid)-poly(vinyl alcohol/polyacrylamide/Zn^2+^ (PAA-PVA/PAM/Zn^2+^) with the tensile strength up to 9.45 MPa and repeatable sensing performance was successfully fabricated by Zheng et al. [22] The nanofibrous organohydrogels were composed of crosslinked PAA-PVA NFMs and PAM network and Zn^2+^ through in situ gelation method (Fig. 5i). As shown in Fig. 5j, the nanofibrous organohydrogels with an AM concentration of 1 mol L^−1^ exhibited a significant increase in stress and elongation at break, reaching 9.45 MPa and 220%, respectively, compared to the nonfibrous organohydrogels, which had a stress of 1.5 MPa and an elongation at break of 43%. Additionally, the nanofibrous organohydrogels can be used at different temperatures of 25, 0, and − 25 °C due to the anti-freezing property of organohydrogels (Fig. 5k). (4) Nanofiber/hydrogel composite materials can be prepared by two-nozzle electrospinning polymer spinning solution and hydrogel precursors on the same collector simultaneously. Ren et al. [30] prepared a nanofibrous hydrogel composite consisting of rigid TPU nanofibers and PVA hydrogel fibers crosslinked with formic acid through two-nozzle electrospinning, then introduced Ag NPs and MXene into it to endow it with sensing capabilities (Fig. 5l). The resulting nanofibrous hydrogel composite exhibits excellent mechanical properties and anti-swelling properties, making it sufficiently capable of effectively monitoring human movements in both air and aquatic environments. Notably, it can accurately identify multidirectional underwater movements and utilize Morse code to achieve intelligent underwater alarms (Fig. 5m, n). The aforementioned nanofiber/hydrogel composite materials exhibit physicochemical similarities with natural biological tissue while exhibiting strong mechanical properties. They open up new horizons for the preparation of novel flexible and stretchable composite sensors.

### Nanofiber/Aerogel Composite Materials

**Fig. 6:**
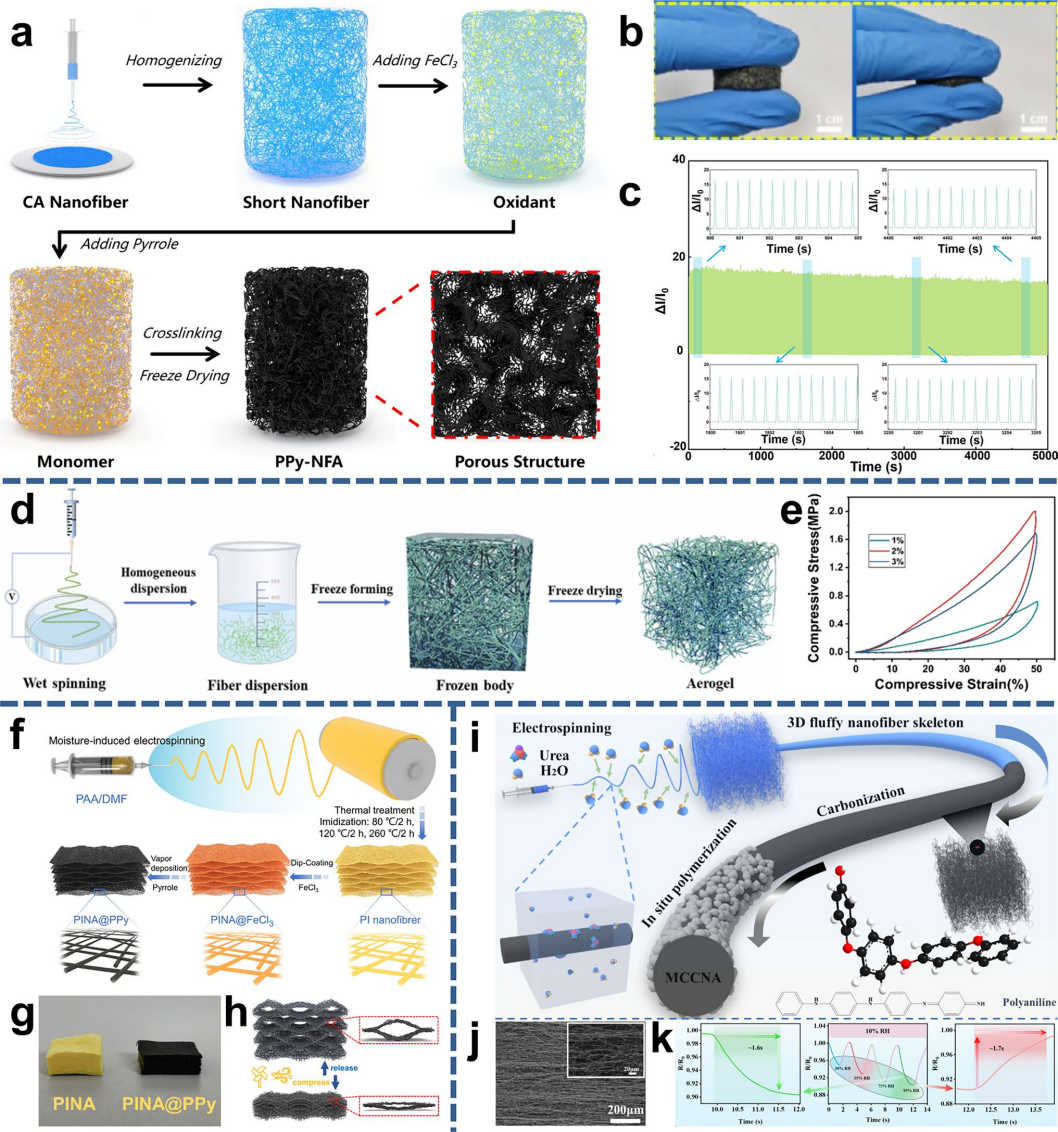
Nanofiber/aerogel composite materials. **a** Preparation process of CA nanofiber composite aerogel. **b** Digital photos of composite aerogel at different compression ratios. **c** Duration test of composite aerogel with over 13,000 cycles; Reproduced with permission from Ref. [19]: Copyright 2021, Elsevier B.V. **d** Fabrication process of the composite aerogel. **e** Compression curves of nanofiber aerogels with different PU concentrations; Reproduced with permission from Ref. [20]: Copyright 2023, Elsevier Ltd. **f** Schematic illustration of the preparation procedure of the PINA@PPy by controlling the relative humidity. **g** Optical images of PINA before and after vapor deposition of PPy. **h** Sensing performance of PINA@PPy pressure sensor; Reproduced with permission from Ref. [33]: Copyright 2024, Elsevier Ltd. **i** Preparation of composite carbon nanofiber aerogel.** j** Interlayer porous structure of the aerogel.** k** Humidity-sensitive performance of aerogel; Reproduced with permission from Ref. [174]: Copyright 2024, Elsevier Ltd

Aerogels are lightweight solid materials characterized by extremely high porosity, and high specific surface area [187]. Compared with traditional aerogels, nanofiber/aerogel composite materials possess additional mechanical flexibility and toughness due to large amount of chemical crosslinking and physical entanglement, which can avoid stress concentration [176]. So far, 3D nanofiber aerogels are mainly fabricated by freeze-drying the obtained crushed 2D NFMs dispersion [10, 20, 188]. The NFMs can be effectively fragmented into short nanofiber structures by homogenizer or ball milling and the length of short nanofibers mainly depends on the characteristic of the material itself, as well as the vigorousness and duration of the homogenization process. To endow the aerogels conductivity, conductive materials can be incorporated in the crushed 2D NFMs dispersion or NFMs during the electrospinning in advance. Qin et al. [19] developed PPy crosslinked nanofiber aerogel (PPy-NFA) by high-speed homogenizer NFMs and freeze-drying method (Fig. 6a). The conductive network was constructed through in situ polymerization PPy on the short CA nanofiber skeleton materials. The nanofiber-based aerogel has an extremely low volume density of 14.67 mg cm^−3^, compressibility of up to 12 kPa, and can endure over 13,000 compression cycles (Fig. 6b, c). Considering the adhesion between nanofibers, binders, including PU and gelatinized starch, are sometimes added to the dispersion [17]. Sun et al. [20] reported a controllable super-elasticity nanofiber/aerogel by introducing PU as bonding agent in a piezoelectric PVDF wet electrospinning precursors, and consequently freeze-drying forming PVDF nanofiber aerogel based nanogenerators (PVPU-NG), as shown in Fig. 6d. The compressive strength of the nanofiber/aerogel was increased to 2 MPa at 50% strain due to the PU would facilitate the formation of a dense network of nanofiber within the aerogel (Fig. 6e).

Another way to prepare 3D nanofiber/aerogel composite material is by direct electrospinning [189]. Relative humidity modulates the charge density of the charged jets, which has an important impact on the direct construction of fluffy nanofiber network structures, i.e., the nanofibers solidify slowly at higher humidity, thus increasing the viscoelastic stress in the NFMs and producing fluffy nanofiber aerogels. Lin et al. [33] proposed a fluffy polyimide nanofibrous aerogel (PINA@PPy) sensor by moisture-induced electrospinning at a relative humidity of 85% and vapor deposition of conductive PPy (Fig. 6f). According to the optical image, the aerogel has a distinct 3D structure and a low density of 0.151 g cm^−3^ (Fig. 6g). When exposed to airflow, normal pressure transfer occurs at the sensor surface and tiny gaps in the nanofiber aerogel are deformed, allowing sensitive detection of airflow at the sensor surface (Fig. 6h). It is worth noting that urea is utilized to induce phase separation by attracting water molecules, causing the charge jets to solidify prematurely in the fluid phase, resulting in the formation of three-dimensional porous nanofibers. Yan et al. [174] fabricated 3D nanofiber aerogels in a one-step process by introducing a structure-functional modifier, urea, into a PAN electrospinning solution, and then subsequently prepared multifunctional conductive carbon nanofiber aerogels (MCCNA) by high-temperature carbonization and in situ polymerization of PANI (Fig. 6i). The fluffy structure is formed by layer-by-layer arrangement of nanofiber layers, and the interlayer porous characteristics provide a convenient space for the transfer of water molecules (Fig. 6j). Due to the different dielectric constants of water and other materials, the adsorption and desorption of water molecules result in a change in electrical resistance, endowing the nanofiber aerogel with humidity variation recognition (10% RH to 95% RH) (Fig. 6k). Three-dimensional nanofiber/aerogel composite materials are well-suited for physical signal detection, offering adjustable flexibility and a porous microstructure.

### Nanofiber/Metal Composite Materials

**Fig. 7:**
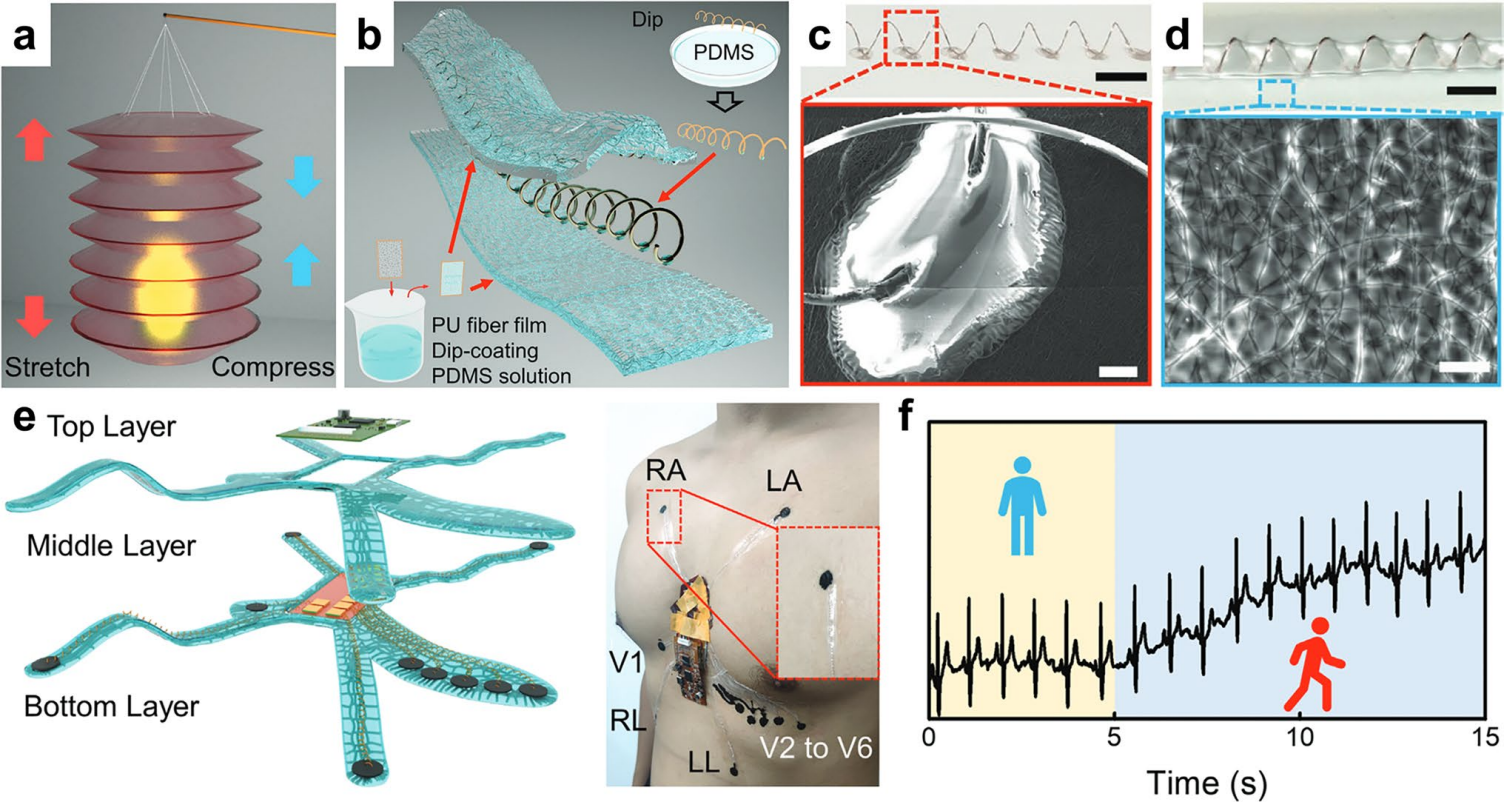
**a** Schematic structure of the accordion lantern. **b** Fabrication of composite materials. **c** Photograph of a copper fiber fixed on PU NFMs by PDMS and SEM image of a PDMS bonding point. **d** Optical photo of an OSHI and SEM image of the PU NFMs with PDMS coating. **e** Assembled inspection systems for electrocardiogram (ECG). **f** Signals are recorded by the system; Reproduced with permission from Ref. [11]: Copyright 2023, Wiley–VCH

Metal exhibits excellent potential for wearable sensors due to their superior conductivity and stability. Nevertheless, the inherent rigidity of metal restricts its broader application in wearable devices. Therefore, integrating metal materials with electrospun nanofibers can improve the comfort of sensors for human signal detection. Nanofiber/metal composite materials not only preserve the metal’s high electrical conductivity but also allow for easier integration of ultrathin nanofibers in epidermal electronic sensors. Li et al. [11] reported the lantern-inspired electrospun PU nanofiber-based metal composite materials (on-skin helical interconnect called OSHI) for electrocardiogram detection, where the metal fiber act as electrical conductors and polymeric membrane with PDMS serves as self-adhesive, light, and robust substrates (Fig. 7a-d). The device was designed as a spider shape based on the electrode positions of 12-lead ECG for human health detection (Fig. 7e). The signal baseline remained stable during standing and sitting, but increased initially and then quickly stabilized when the volunteer started jogging, demonstrating reliable and sensitive signal detection (Fig. 7f).

NFMs and metal materials exhibit complementary properties, with nanofibers enhancing the comfort of metal materials, while metal materials augment the conductivity and support of NFMs. However, there are limited instances of nanofiber/metal composite materials being used for flexible sensors, in order to solve the limitation of the rigidity property of metal materials in the human epidermis. Dong et al. [190] used finer metal filaments for weaving to form flexible stretchable and bendable devices. Nevertheless, this approach still poses challenges for ensuring optimal comfort on the skin surface. Additionally, the metal structure can provide support for the fluffy fiber structure, which can avoid performance degradation due to collapse. By combining the unique properties of nanofiber membranes and metal materials, these composite materials can offer enhanced functionalities and capabilities compared to traditional materials used in flexible sensors.

In this section, we discussed the different types of nanofiber-based composite materials (nanofiber/hydrogel, nanofiber/aerogel, and nanofiber/metal composite materials) for monitoring human signals and summarize the advantages and disadvantages of the fabrication methods of different composite materials in Table 5. Different structures owned by exact materials could always be chosen to form an enhancing performance. For nanofiber/hydrogel composite materials, the topology and physical entanglement of the nanofibers effectively enhance the mechanical properties and durability of the hydrogel, while the excellent biocompatibility and skin-friendliness of the hydrogel make the composites play an important role in the field of flexible electronic devices. Meanwhile, solving the problem of decreased sensing performance of nanofiber/hydrogel composite materials due to water loss in the natural environment is the key to the sensing application. The studies on nanofiber/aerogel composite materials have shown that the flexibility of the composite materials can be achieved by transferring stress and deformation by incorporating flexible nanofibers. Thus, using the flexible chains formed by the nanofibers, a continuous micro-network structure can be achieved, which plays an important role in determining the mechanical and sensing performance of the aerogel composites. The novel composite materials prepared by combining nanofiber membranes with flexible qualities and highly conductive hard metals have promising applications in the field of flexible electronics. However, the combination at the joint of flexible and rigid materials is still a challenging problem. Many novel materials are being explored to create innovative composite materials with NFMs, which are expected to enhance performance in the sensing area.

**Table 5:** Advantages and disadvantages of different nanofiber-based composite materials

Material	Methods	Advantages	Disadvantages
Nanofiber/hydrogel	Spin coating	Uniform and thin hydrogel layer; controlled thickness	Insufficient interfacial bond strength; material waste
	Drop coating	Simple operation; controlled thickness; localized drop coating	Insufficient interfacial bond strength; uneven coating
	Homogenization gelation	Improved interface integration; improved mechanical property	Agglomeration of nanofibers; preparation complexity
	In situ gelation	Stronger bonding of the two materials; in situ adjustment	Process complexity
	Two-nozzle electrospinning	Compounding of multiple materials in one step	Uneven hydrogel deposition due to electric field interference
Nanofiber/aerogel	Homogenization freeze-drying	Enhanced mechanical strength	Long preparation time
	Direct electrospinning	Quickly construction of a fluffy structure	Requires subsequent conductivity

## Application of Electrospun Nanofiber-Based E-Skin for Physical, Physiological, Body Fluid, and Multi-Signal Monitoring

Electrospun nanofibrous composite sensors have attracted considerable research interest due to their numerous advantages, including high accuracy, superior skin conformability, and outstanding flexibility and elasticity. Additionally, their excellent wearability, coupled with the integration of encapsulation technology, significantly enhances their portability and facilitates continuous monitoring capabilities. This section presents a summary of the advancements made in three major areas: physical signals, physiological signals and body fluid signals, along with their integrated multi-signal applications.

### Physical Signals

**Fig. 8:**
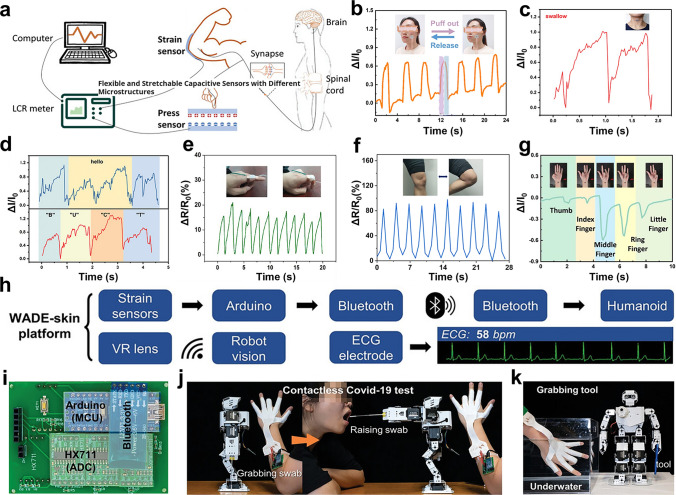
Physical signals sensing via electrospun nanofiber composites sensors. **a** Schematic illustration of signal conversion in human feedback system and E-skin; Reproduced with permission from Ref. [191]: Copyright 2021, Wiley–VCH. **b** Worn at the cheek to detect facial movements; Reproduced with permission from Ref. [23]: Copyright 2023, Royal Society of Chemistry. **c** Worn on the throat to detect swallow. **d** Worn on the throat to detect and distinguish articulation; Reproduced with permission from Ref. [10]: Copyright 2020, Wiley–VCH. **e** Wearing on the finger joints to detect joint flexion; Reproduced with permission from Ref. [192]: Copyright 2022, Elsevier. **f** Wearing on the knee joints to detect joint flexion; Reproduced with permission from Ref. [8]: Copyright 2022, Springer Nature. **g** Wearing on the finger joints to distinguish fingers bending signals; Reproduced with permission from Ref. [19]: Copyright 2022, Elsevier. **h** System integration platform of WADE-skin.** i** Digital images of printed circuit board (PCB) used for processing and transmitting the signals. **j** Digital images of the contactless nucleic acid test by controlling the robot remotely. **k** Digital images showing underwater control of the humanoid robot for grabbing tools; Reproduced with permission from Ref. [193]: Copyright 2023, Wiley–VCH

Electrospinning nanofiber-based composite sensors have shown significant promise in detecting a wide range of physical stimuli, which can continuous and unobtrusive monitoring of human movements and mimic the sensing mechanism of human skin through converting physical signals into electronic signals (Fig. 8a) [191]. The detection of varying levels of body movements can be achieved by either attaching or seamlessly integrating sensors directly onto the human body. A lightweight nanofiber composite aerogel was prepared for detecting tiny muscle movements in facial floats, which was achieved by blending homogenized electrospun poly(sulfonamide) (PSA) and PAN nanofibers with rGO, followed by a careful freeze-drying process [23], and the remarkable consistency between the alterations in resistance and the corresponding movements can be observed (Fig. 8b). Cao et al. have successfully fabricated nanofiber reinforced graphene aerogel (aPANF/GA) aerogels by integrating alkaline treated PAN electrospun nanofibers with rGO, followed by freeze-drying process, which can detect signals generated by swallowing and different word pronunciations in the throat (Fig. 8c, d) [10]. The shape of the signal is altered when the word “hello” is spoken at varying speeds, and different signals are observed for different letters.

In addition to detecting subtle physical signals, electrospinning nanofiber-based sensor is also capable of detecting large-scale human movements. An organic anti-freeze hydrogel developed by integrating electrospun PU NFMs with PAM-grafted gelatin hydrogel has been reported to detect the finger bending movements (Fig. 8e) [192]. When finger bends regularly from down to up, the response resistance changes simultaneously, indicating promptness and precision detection of finger movements. Furthermore, an advanced hybrid ionic skin has been developed through embedding PU nanomesh within a flexible supramolecular ionic matrix for accurately detecting knee bending movement (Fig. 8f) [8]. PPy-NFA nanofiber aerogels were fabricated via in situ polymerization of pyrrole and subsequent electrospinning of cellulose acetate nanofibers, followed by a freeze-drying technique. Due to the different degrees of contraction of different flexor tendons, the aerogels demonstrate an impressive ability to distinguish the bending signals generated by different fingers, providing unmatched precision in motion detection (Fig. 8g) [19].

Wireless signal transmission represents a pivotal development trajectory within the realm of wearable devices, underpinning their advancing capabilities and improving convenience into daily life [194]. Chen et al. presented a wet-adaptive electronic skin (WADE-skin) that comprises a next-to-skin PAAND wet-adhesive hydrogel fibrous layer, a next-to-air waterproof fibrous layer, and a stretchable and permeable LM electrode layer. In particular, PAAND fibers form a thin conformal hydrogel layer that uniformly coats the skin surface while simultaneously penetrating and encapsulating adjacent SBS fibers. This structural integration significantly enhances the interfacial adhesion between the fibrous network and the polymer matrix. By integrating the WADE-skin with VR headsets, printed circuit boards, and Bluetooth systems (Fig. 8h-i), the sensor could wirelessly control biomimetic robots to grasp throat swabs and perform non-throat swab tests (Fig. 8j) [193]. Additionally, they allow for remote control of the robot’s underwater grasping tools, which holds significant potential for underwater applications such as underwater fixation, transportation, and mineral exploration (Fig. 8k).

**Fig. 9:**
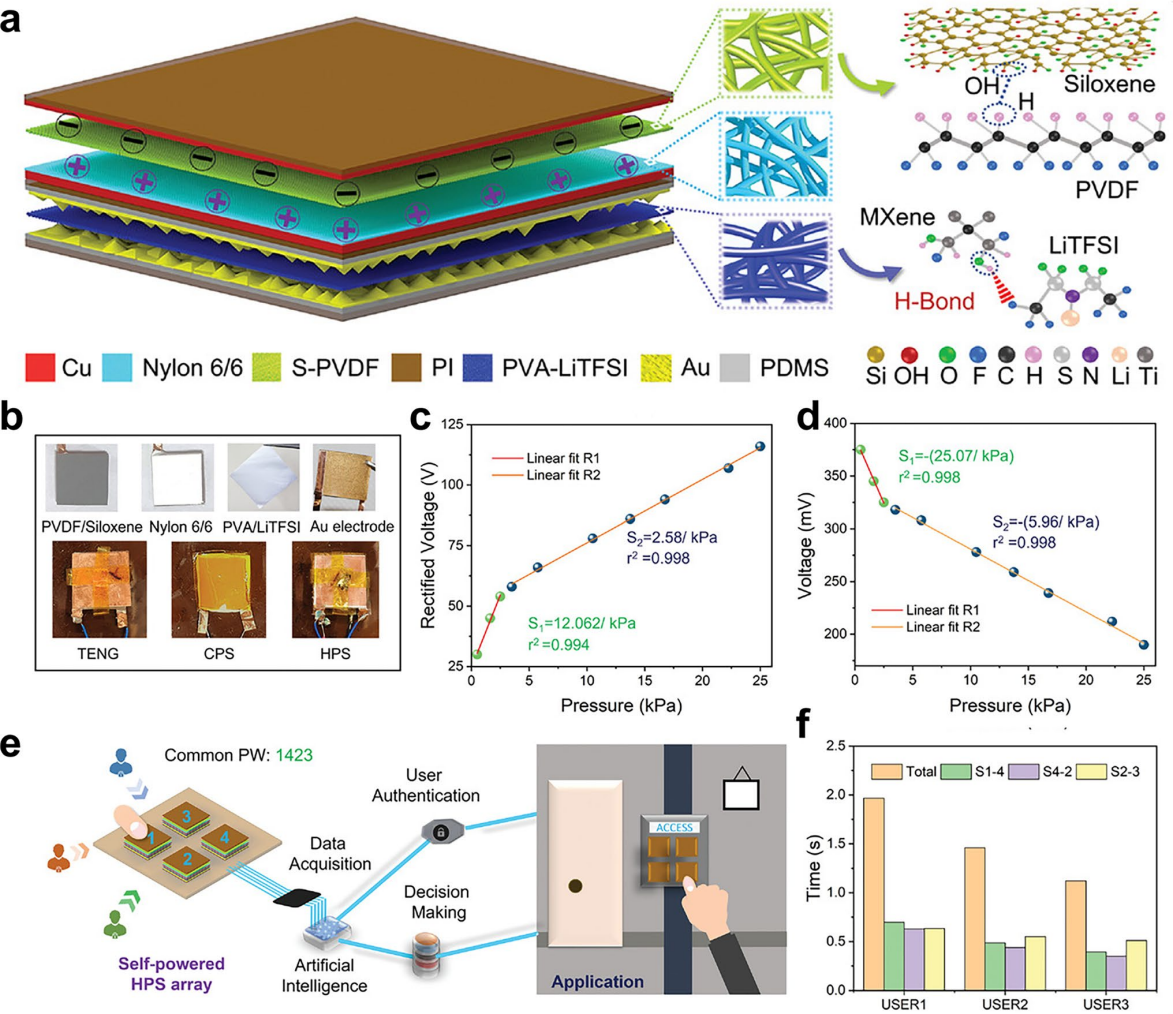
Physical signals sensing via self-power electrospun nanofiber composites sensors. **a** 3D Schematic illustration of the TENG and self-powered hybrid pressure sensor (SP-HPS). **b** Photographs of different components of the SP-HPS. **c** Sensitivity curve of SP-HPS under dynamic pressure conditions. **d** Sensitivity curve of SP-HPS under static pressure conditions. **e** Architectural framework of an intelligent user authentication system designed for personalized security in electronic access control, utilizing artificial intelligence. **f** Comparative analysis of user recognition accuracy (expressed as a percentage) for three individuals using the common access code “1432” across three distinct scenarios, along with the calculated average accuracy. Reproduced with permission from Ref. [196]: Copyright 2021, Wiley–VCH Copyright 2022, Wiley–VCH

In conjunction to physical monitoring using passive sensing technologies, their self-powered alternatives based on triboelectric and piezoelectric principle are emerging rapidly [124, 195–198]. These innovative energy-harvesting systems leverage the inherent mechanical energy from body movements, respiration, or even blood flow to generate electrical signals without requiring external power sources, thereby addressing critical limitations of conventional battery-dependent devices. Trilochan et al. developed a triboelectric nanogenerator (TENG) using a 2D siloxene-PVDF (S-PVDF) composite nanofibrous membrane, integrated with a capacitive pressure sensor (CPS) to create a hybrid sensor (SP-HPS) (Fig. 9a, b) [196]. Experimental results show that the SP-HPS demonstrates high sensitivity in both dynamic (12.062 V kPa^−1^ at < 3 kPa; 2.58 V kPa^−1^ at 3–25 kPa) and static (25.07 mV kPa^−1^ at < 3 kPa; 5.96 mV kPa^−1^ at 3–25 kPa) pressure sensing. This enables precise detection of pressures from light touches to higher forces (Fig. 9c, d). To further validate its application potential, the research team constructed a user authentication system using an SP-HPS array and neural networks, achieving near-perfect recognition accuracy across static, dynamic, and hybrid sensing modes (Fig. 9e, f). Additionally, to address the critical issue of limited dipole alignment in piezoelectric materials, Zhang et al. innovatively designed and fabricated a spatially confined MXene/PVDF nanofiber composite membrane [197]. The spatial confinement of MXene/PVDF nanofibers actively induces optimal alignment of the –CH_2_–/–CF_2_– dipoles in PVDF, significantly enhancing spontaneous polarization and improving the piezoelectric performance. The fabricated MXene/PVDF nanofiber-based piezoelectric device demonstrated remarkable improvements, generating voltage and current outputs that were 3.97 times and 10.1 times higher, respectively, compared to pure PVDF nanofibers. This breakthrough not only enhances the efficiency of piezoelectric materials, but also enables practical applications in monitoring various human body movements and harvesting energy from them.

Building on these advancements in energy-autonomous sensing, the development of high-performance nanofiber-based sensors for physical signal detection necessitates a systematic optimization of material properties to meet stringent operational demands. For physical signal detection, nanofibers must exhibit high flexibility, sensitivity, and cyclic stability. Enhanced stretchability is realized through intrinsically elastic polymers combined with electrospinning techniques such as dual-electrode twisting [199], photolithography [200], and pre-stretching wrinkling [201], which enable the fabrication of interlaced fiber yarns with superior tensile properties, patterned membranes for large deformations, and wrinkled structures for effective stress dispersion. Sensitivity improvements are achieved by tailoring sensing-layer structures via near-field electrospinning [47, 51], specialized collectors [202, 203], and adjustable drum speeds [204], allowing precise material deposition and oriented fiber mats that enhance uniaxial sensing capabilities. Additionally, surface charge control [205] and sacrificial template methods [206, 207] create micro-patterned surfaces to boost pressure sensor sensitivity. To ensure long-term reliability, cyclic stability is improved through material modifications including blending and grafting for self-healing properties and structural optimizations such as pre-stretching and surface wrinkling for stress dispersion. These strategies collectively enable robust, high-performance nanofiber sensors suitable for complex and prolonged use.

Electrospinning technology enables the preparation of nanofibers with high specific surface areas and fine diameters, significantly enhancing the sensitivity of the sensors in capturing physical signal variations. Through rational structural design, electrospun nanofibrous composite sensors can detect multiple physical signals according to specific requirements, meeting the demands of different application scenarios. However, flexible sensors still face multiple challenges in physical signal sensing. Firstly, achieving high sensitivity across a wide dynamic range remains a critical issue, as high sensitivity often conflicts with a narrow dynamic range. For instance, highly sensitive pressure sensors may saturate under large strains, while sensors with a wide dynamic range may fail to detect minor deformations. Secondly, real-time monitoring requires sensors to exhibit fast response capabilities and low power consumption to meet the endurance requirements of wearable devices. However, high-power consumption may limit the continuous operation time of such devices. Additionally, repeated mechanical deformation can lead to material fatigue or performance degradation, affecting long-term stability and durability. For example, flexible sensors may experience conductive layer fractures after multiple stretching cycles, resulting in signal distortion. Addressing these challenges is essential for achieving high-precision and reliable physical signal monitoring. To better understand the current state of electrospun E-skin development and its performance characteristics, Table 6 provides a comprehensive summary of the core performance parameters of electrospun nanofiber-based composite materials in physical signal sensing and detection in recent years.

**Table 6:** Performance evaluation of electrospun nanofiber-based composite materials on physical signals

Materials	Maximum sensitivity	Working range	LOD	SNR	Stability	Other performances	Refs
[Tb(TTA)_2_ /GE]/[PANI/GE]	4.24	10%-30%		–	–	Luminescent	[104]
P(AMPS-co-AAm)/ /PVA/TPU	1.58	0–100%	0.1%	–	4000 cycles	Breathable; anti-freezing	[26]
PVA/SA/TA/TPU	1.2	0–100%	–	–	100 cycles	Self-adhesive; antibacterial	[184]
PVDF-HFP/IL	6.44	0–150%	1%	–	–	High mechanical	[208]
PAM/GE/PU	3.87	0–600%	1%	–	250 cycles	Antifreezing	[192]
CA + TPU/PPy	33.56 kPa^−1^	0–24 kPa	30 Pa	–	5500 cycles	High mechanical	[63]
PW + PPy/Pg@Ag	27.75 kPa^−1^	0–440.6 kPa		–	5000 cycles	Photothermal conversion	[24]
PAAS/GO	18.55 kPa^−1^	0–7.5 kPa	2 Pa		3000 cycles	–	[209]
PI/PPy	0.42 kPa^−1^	0–25 kPa	20 Pa	–	5000 cycles	Sound detection and recognition	[33]
PSNA/rGO	32.85 kPa^−1^	0–24.97 kPa	–	–	3500 cycles	Temperature resistant	[23]
PU/Nylon/Mica	0.082 kPa^−1^	0–10 kPa	–	51.9 dB	5000 s	Object recognition	[210]
PVDF-BTO/Ni	2.62 ± 0.11 V kPa^−1^	0–9 kPa	–	37 dB	2500 s	Washable	[211]

### Physiological Signals

Based on controllable mechanical properties, high-performance stand-alone or integrated sensors based on electrospun nanofibers could accurately capture and visualize real-time physiological information pertaining to health, transforming human body electrical signals into comprehensible diagrams such as ECG, EMG, EOG, and EEG, thereby offering invaluable insights into an individual’s physiological state.

**Fig. 10:**
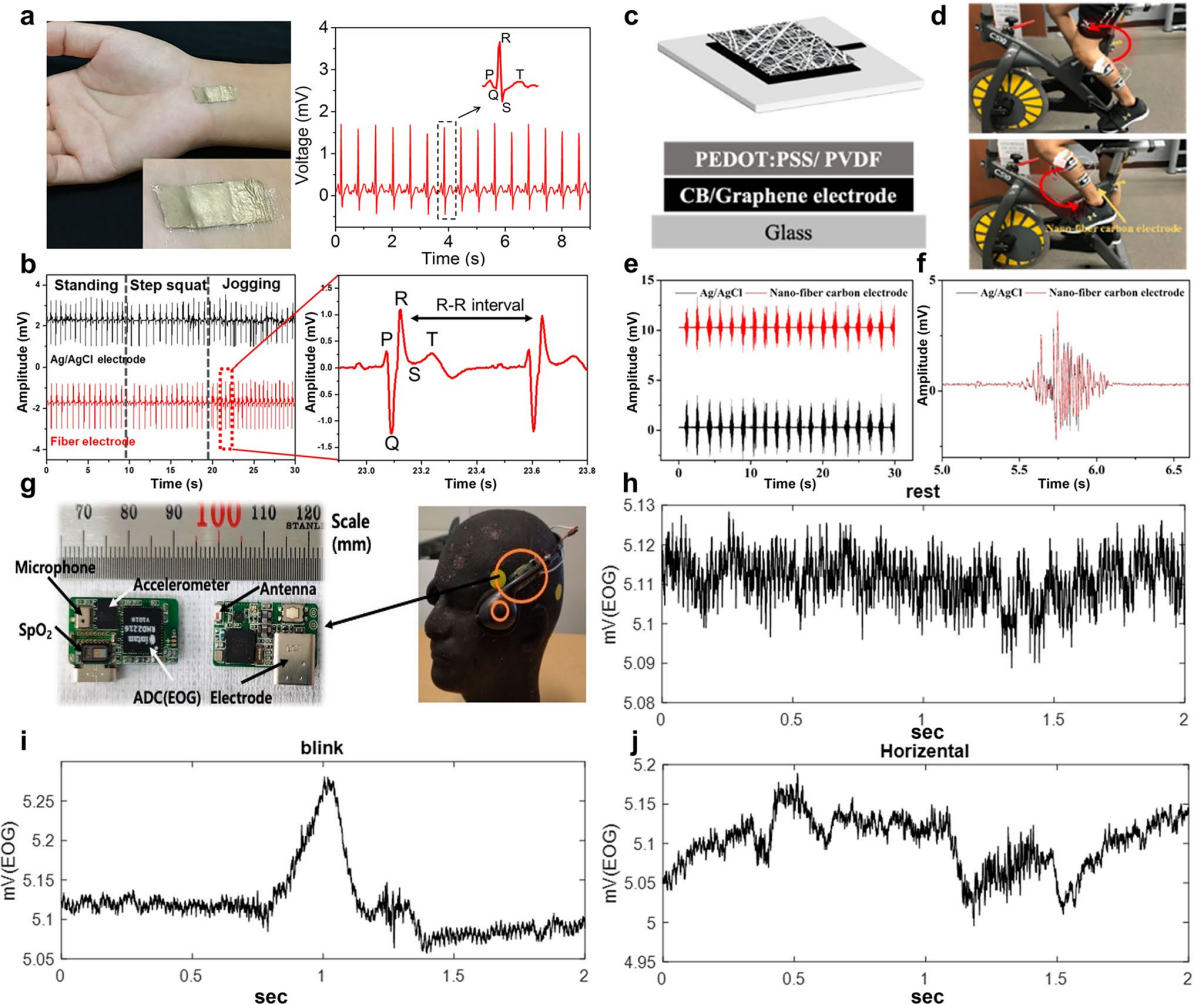
Physiological signals sensing via electrospun nanofiber composites sensors. **a** Photographs of the hydrogel-elastomer hybrid mounted on the wrist and ECG signals recorded by the hybrid electrodes; Reproduced with permission from Ref. [212]; Copyright 2020, Wiley–VCH. **b** ECG signals and magnified plots of signals obtained using Ag NPs/rGO/PEDOT:PSS/TPU composite nanofiber electrodes and Ag/AgCl electrodes; Reproduced with permission from Ref. [109]: Copyright 2023, Springer Nature. **c** Schematic diagram of the CB/rGO/PEDOT:PSS/PVDF nanofiber composite electrode. **d-f** Electrodes were attached to the calf and EMG signals were generated from walking; Reproduced with permission from Ref. [213]: Copyright 2023, Springer Nature. **g** Wearable EOG hardware and **h-j** eye movement EOG pattern of “rest,” “blink,” and “horizental” from one channel EOG system with the electrospun electrodes assembled on a headset; Reproduced with permission from Ref. [54]; Copyright 2023, MDPI

Pan et al. have reported the development of a robust hydrogel-elastomer hybrid material, achieved by infiltrating the hydrogel precursor into an electrospun TPU nanofiber network, followed by thermal curing. Prior to the infiltration of the hydrogel precursor, a gold nanofilm was deposited onto the TPU surface via thermal evaporation, imparting conductivity to the composite [212]. The prepared samples exhibit remarkable conformability, enabling them to adhere closely to the wrist. When utilized for ECG signal recording, all the P waves, Q waves, R waves, S waves, and T waves can be distinctly discerned (Fig. 10a). These signals are of paramount importance in assessing heart rate and various cardiac arrhythmias. The Ag NPs/rGO/PEDOT:PSS/TPU composite nanofiber electrodes prepared via immersing the electrospun TPU NFMs into the Ag NPs/rGO/PEDOT:PSS nanohybrids conductive paste and sonication were fixed to the chests region to detect breathing patterns [109]. During standing and walking, the ECG signals generated by commercial Ag/AgCl electrodes were almost identical to those generated by the fiber electrodes. However, during jogging, the custom-designed fiber electrode maintained a high degree of stability, showing a clearer and more distinct P-Q-R-S-T peaks, accompanied by significantly less noise in the ECG data (Fig. 10b).

EMG refers to the recorded bioelectrical waveforms of skeletal muscles obtained using electromyography equipment. This recorded waveforms are invaluable in diagnosing neuromuscular disorders and in controlling prosthetic devices [214, 215]. Huang et al. electrospun PEDOT:PSS/PVDF nanofibers onto a substrate composed of CB and rGO (CB/rGO/PEDOT:PSS/PVDF), crafting a sophisticated nanofiber carbon electrode that excels in monitoring EMG signals with exceptional precision and sensitivity (Fig. 10c) [213]. When the CB/rGO/PEDOT:PSS/PVDF nanofiber composite electrode was fixed to the calf, the EMG signals measured by the fiber composite electrodes was similar to those measured by the Ag/AgCl electrodes. By further assessing the amplified EMG signals, it is evident that the signal curves generated by the CB/rGO/PEDOT:PSS/PVDF nanofiber composite electrode closely align with those commercial Ag/AgCl electrodes, definitively demonstrating its outstanding reliability (Fig. 10d-f).

**Fig. 11:**
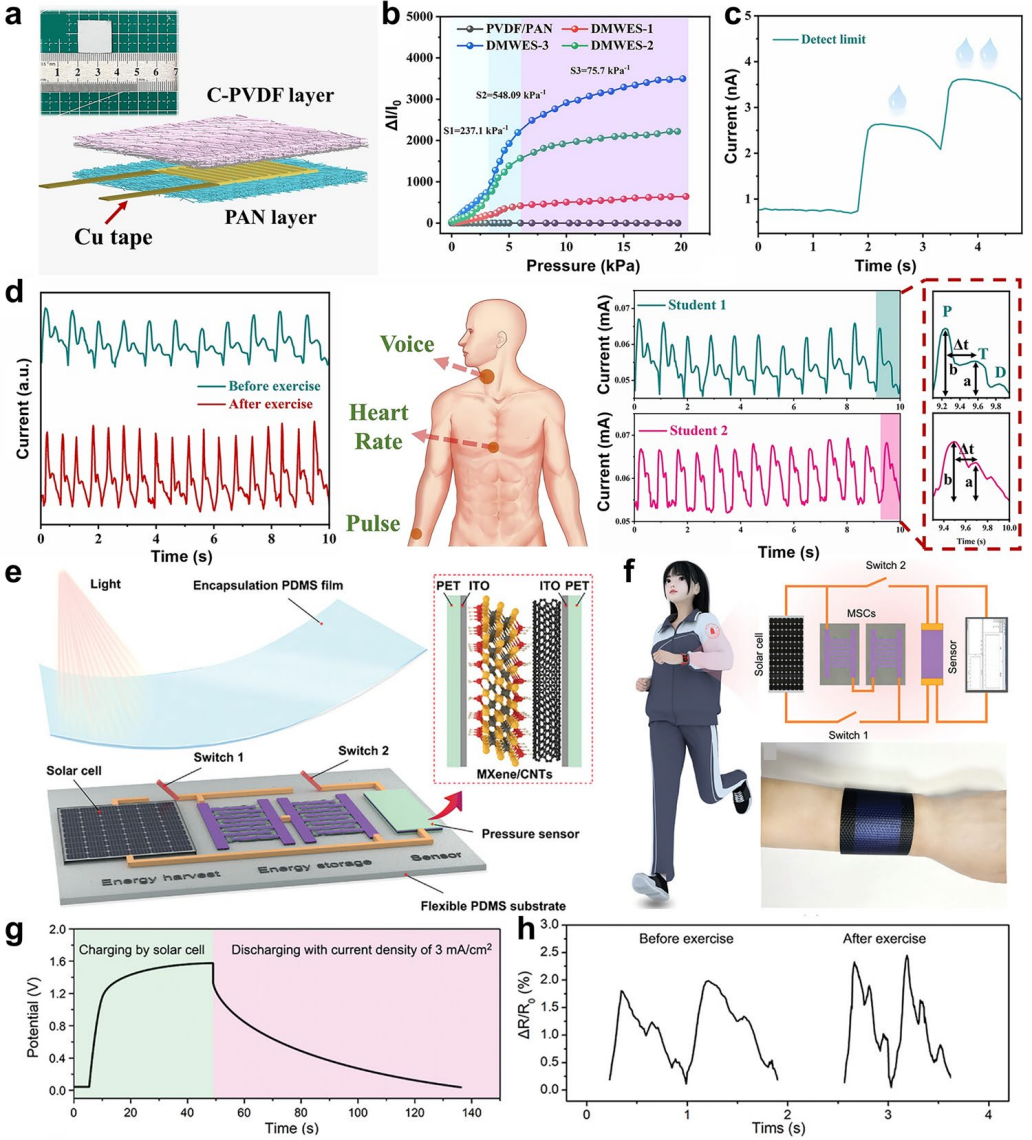
Physiological signals sensing via self-power electrospun nanofiber composites sensors. **a** Schematic illustration of the DMWES as pressure sensors. **b** Sensitivity performance curve of the DMWES. **c** Detection limit of the DMWES. **d** Wrist pulse signals captured from a 28-year-old male before and after exercise, as well as from two students. Reproduced with permission from Ref. [216]: Copyright 2023, The Authors. **e** Integrate solar cell, micro-supercapacitor, and pressure sensor to obtain self-powered sensor. **f** Schematic illustrate the self-powered flexible sensor system for real-time pulse single detection. **g** Charging and discharging curves of the self-powered flexible sensor, charged by a solar cell array and discharged at a current density of 3 mA cm^−2^. **h** Real-time monitoring of human pulse signals before and after exercise. Reproduced with permission from Ref. [217]: Copyright 2023, Wiley–VCH

EOG enables the measurement of the retina’s action potential, assessing an individual’s level of alertness and eye movements during drowsy driving. Moon et al. [54] developed a novel hybrid semidry electrode with an electrolyte gel impregnated inside the microfiber core based on melt electrospinning for EOG detection (Fig. 10g). Subsequently, a wireless wearable device designed for capturing EOG signals was devised, which could collect data on eye relaxation, blinking and horizontal movement electrical signals by placing a pair of semidry electrodes mounted on a small headset on either side of the eye (Fig. 11h-j). It is noteworthy that this electrode boasts an impedance that is 3.5 times lower than traditional dry electrodes, while maintaining a stable impedance level, showing minimal drift even with extended wear.

Currently, the application of electrospun nanofiber composite materials in the field of EEG signal acquisition remains largely unexplored, representing a significant research gap with considerable potential. The unique characteristics of electrospun nanofiber composites, including their high specific surface area, tunable porous structures, and exceptional flexibility, offer novel potential solutions to critical challenges in EEG monitoring.

Furthermore, self-powered electrospun nanofiber composite sensors hold significant promise in the field of physiological signal detection [128, 217]. Zhi et al. developed a directional moisture-wicking electronic skin (DMWES) using electrospinning technology (Fig. 11a) [216]. The DMWES membrane demonstrates outstanding comprehensive pressure-sensing capabilities, characterized by high sensitivity (with a maximum sensitivity of 548.09 kPa^−1^) and an ultralow detection limit, enabling it to detect the pressure of a single water droplet (approximately 5 Pa) (Fig. 11b, c). Thanks to its exceptional sensing performance, the DMWES can be attached to the wrist for real-time human pulse monitoring. It is capable of distinguishing variations in pulse frequency and intensity before and after physical activity, as well as accurately detecting the P, T, and D waves in the pulse waveform (Fig. 11d).

In addition to utilizing self-powered electrospun nanofiber composite sensors for precise physiological signal detection, the integration of power sources with sensing systems offers a novel approach for monitoring physiological signals. Wang et al. developed a micro-supercapacitor (MSC) based on Ti_3_C_2_T_x_/CNTs@SEBS/CNTs thin films and integrated it with a solar cell, demonstrating its feasibility for biosensing applications [217]. As shown in Fig. 11e, two series-connected MSCs serve as energy storage devices, charging the solar cell while simultaneously powering a flexible sensor designed to detect human pulse. Furthermore, the electrical performance of the integrated MSC under solar cell charging and constant current density discharging conditions was evaluated, with the results presented in Fig. 11f, g. Before charging with the solar panel, the system’s open-circuit potential was 0.06 V, which increased to 1.6 V within 45 s under standard solar illumination. As illustrated in Fig. 11h, the self-powered sensor system successfully achieved real-time monitoring of human pulse before and after exercise. The experimental results revealed pulse rates of 75 bpm and 122 bpm before and after exercise, respectively, with consistent heartbeat waveform characteristics. This integrated system highlights its significant potential in the field of wearable health monitoring.

While these integrated self-powered systems demonstrate significant advancements in physiological monitoring, achieving clinically reliable signal detection necessitates fundamental improvements in the core properties of nanofiber-based sensors themselves. In the field of physiological signal detection, nanofibers must meet fundamental requirements such as low impedance, SNR, and biocompatibility. To reduce surface impedance, commonly employed techniques include loading metals or conductive materials [109, 158, 218–220], in situ polymerization of conductive coatings [221], and chemical vapor deposition (CVD) [222]. These methods not only significantly lower surface impedance, but also maintain the flexibility of nanofibers while optimizing their conductive properties. Increasing the contact area between sensors and the skin significantly enhances the SNR, a critical strategy for optimizing physiological signal detection. Fabrication methods such as electrospraying enable the development of ultrathin flexible sensors that conform tightly to the skin, minimizing signal attenuation [158]; hydrogel interfacial layers that increase the effective contact area and reduce interfacial impedance [21]. These approaches not only enhance signal transmission performance but also ensure flexibility, biocompatibility, and wearer comfort, providing robust technical support for high-performance physiological signal detection. To improve the biocompatibility of sensors, key strategies include the use of natural polymers and the development of implantable piezoelectric sensors [223]. The latter exhibit unique advantages due to their self-powered nature: by eliminating the need for external power modules, they simplify integrated circuit (IC) designs, thereby reducing device footprint and mitigating potential thermal/chemical risks. This approach not only enhances biosafety but also improves biocompatibility by minimizing foreign body reactions and ensuring long-term operational stability, providing robust technical support for chronically implantable medical devices.

Electrospun nanofiber composite electrodes or conductors, with their excellent flexibility, strain sensitivity, and cyclic tensile stability, play a crucial role in monitoring human physiological signals such as EOG, ECG, and EMG. However, flexible sensors still face multiple challenges in physiological signal sensing. The high electrode–skin contact impedance particularly affects low-frequency signal detection, requiring development of low-impedance interface materials or optimized surface morphology. The inherently weak physiological signals are vulnerable to environmental noise including motion artifacts and electromagnetic interference, necessitating integrated shielding layers or advanced signal processing algorithms to improve SNR. Long-term wearability issues such as performance degradation demand solutions like self-healing materials or replaceable electrode modules. Furthermore, relative electrode–skin displacement during motion introduces additional noise, calling for improved flexibility/adhesion designs or motion compensation algorithms. Addressing these challenges is essential for achieving reliable, high-precision physiological monitoring. To better understand the current state of electrospun E-skin development and its performance characteristics, Table 7 provides a comprehensive summary of the core performance parameters of electrospun nanofiber-based composite materials in physiological signal sensing and detection in recent years.

**Table 7:** Performance evaluation of electrospun nanofiber-based composite materials on physiological signals

Materials	Conductivity	SNR	Stability	Applications	Other performances	Refs
PVA + PU/Au	1 Ω cm^−2^	–	–	EMG	Ultrathin	[21]
TPU/Ag NPs + rGO + PEDOT:PSS	1.3 × 10^1^ Ω cm^−2^	–	–	ECG/EMG	Self-healing	[109]
TPU + PP/SLM	1.4 × 10^7^ S m^−1^	–	–	ECG	Breathable; waterproof	[224]
PVDF/CB + rGO + PEDOT:PSS	2.5 × 10^1^ Ω sq^−1^	–	3000 cycles	ECG/EMG	–	[213]
PVDF/Ag NPs + CB	4.1 × 10^1^ Ω sq^−1^	–	5000 cycles	ECG/EMG	–	[220]
TPU + PAM + Ca-Alg/Au	40.9 ± 6.3 Ω sq^−1^	17.8 dB	–	ECG/EMG	Self-adhesive	[212]
PU/P(AAm-co-AA)/HA	–	–	–	ECG/EMG	Self-healing	[8]
SBS + PAAND/EGaLn	34,000 S cm^−1^	–	–	ECG	Adhesive	[193]

### Body Fluid Signals

The development of personalized wearable health monitoring devices, which could be used to analyze the components and proportions of bodily fluids (such as sweat [225, 226], saliva [227–229], urine [230, 231], and blood [232, 233]), is crucial for providing individualized diagnosis and treatment [226, 234]. By collecting and analyzing body fluid metabolites signals (such as lactic acid [235, 236], glucose [235–237], pH [238, 239], ionic [236, 240], enzyme [231], adenosine triphosphate [241], and cortisol [242] among others), abnormalities can be detected at an early stage of diseases, enabling early intervention and treatment, thereby improving cure rates and survival rates.

**Fig. 12:**
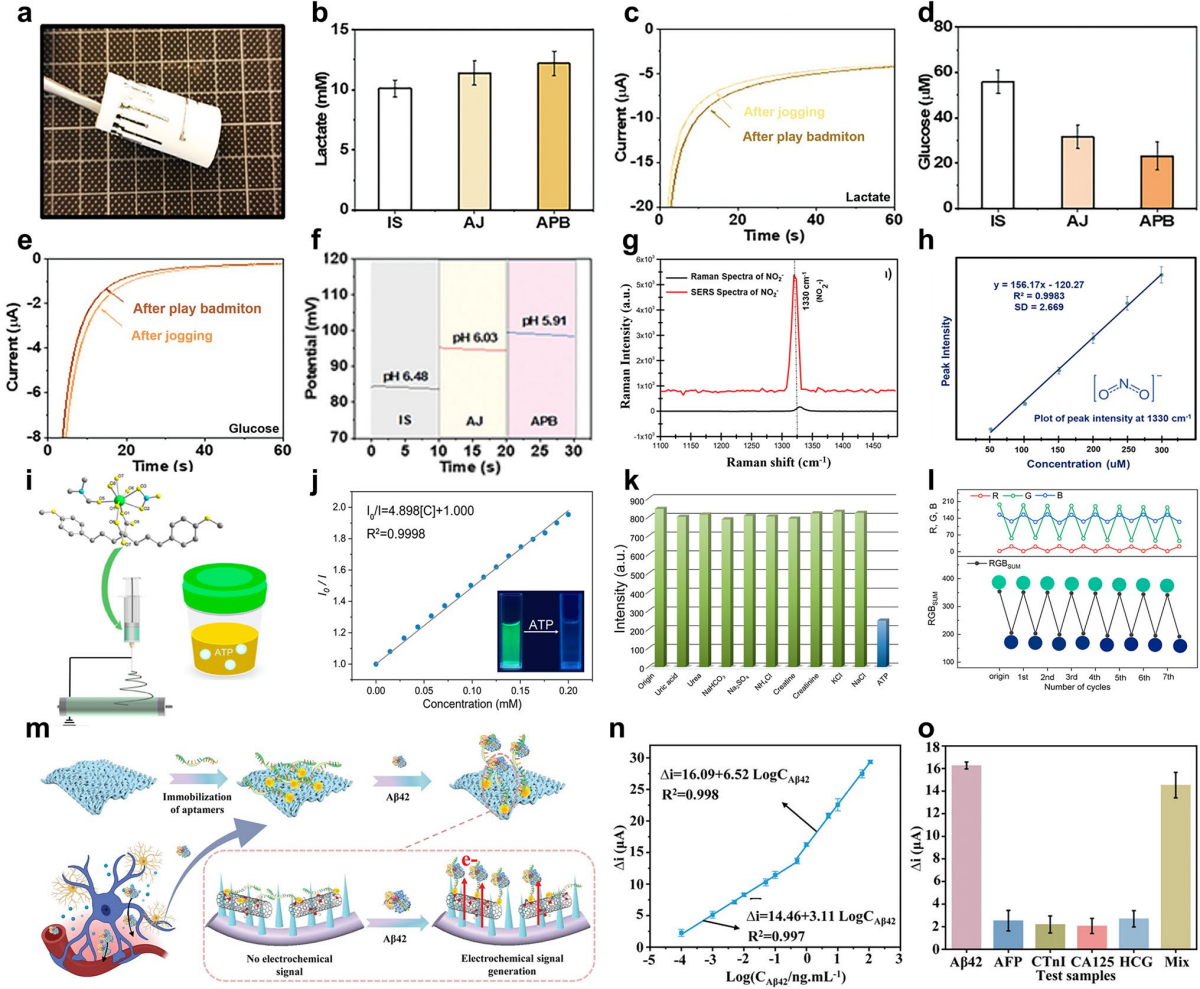
Body fluid sensing using electrospun nanofiber composite sensors. **a** Digital image of the Janus electrospun membrane-based bionic sensing skin. **b-c** Concentration changes of lactate acid in sweat and before and after sports. **d-e** Changes of glucose concentration in sweat and corresponding response curves before and after sports. **f** Changes of pH in sweat before and after sports (IS: initial state; AJ: after jogging; APB: after playing badminton); Reproduced with permission from Ref. [235]: Copyright 2024, Wiley–VCH. **g** Nitrite Raman/SERS spectra using Ag@HCNF substrates. **h** Relationship curve between the peak intensity of nitrite and its concentration; Reproduced with permission from Ref.[229]: Copyright 2024, The Royal Society of Chemistry. **i** Schematic of Tb-BPA/PAN nanofiber membrane for urinary ATP detection. **j** Linear quenching response at 545 nm (inset: UV-induced luminescence changes). **k** T Luminescence intensity with various urine components (0.5 mM each). **l** R, G, B, and RGB sum values for the Tb-BPA-modified luminescent PAN nanofiber composite emission were recorded over 7 cycles. Reproduced with permission from Ref. [241]: Copyright 2024, American Chemical Society. **m** Aβ42 detection mechanism of PA/PANI-CNTs aptasensor. **n** Current response vs Aβ42 concentration.** o** Selectivity evaluation of the aptasensor for 1 ng mL^−1^ Aβ42 in the presence of 100 ng mL^−1^ interfering biomarkers. Reproduced with permission from Ref. [233]: Copyright 2024 The Authors

Shi et al. created a Janus electrospun membrane-based intelligent wearable system that mimics human skin in structure and possesses superior mechanical properties for detecting changes in lactic acid concentration, glucose concentration, and pH levels in sweat before and after jogging, and playing badminton (Fig. 12a). The fabrication of a stable nanofiber welding structure was achieved through solvent welding technology, significantly enhancing the membrane system’s mechanical strength, interlayer bonding force, and overall stability. At the initial state, the concentration of lactic acid and glucose in sweat were measured at 10.1 mM and 55.8 μM. Following jogging, the lactic acid concentration in sweat increased to 11.4 mM. Compared to jogging, playing badminton is a relatively intense activity, resulting in a higher production of lactic acid in sweat. After 30 min of badminton, the lactic acid concentration rose to 12.1 mM (Fig. 12b, c) [235]. In comparison, due to the consumption of glucose during jogging, and playing badminton, the glucose concentration in sweat decreased to 31.6 μM after jogging and 23.1 μM after playing badminton (Fig. 12d, e). Furthermore, the decrease in pH value from 6.48 to 5.91 in sweat after playing badminton provides additional evidence for the further increase in lactic acid concentration (Fig. 12f). These signal variations of these metabolites were successfully captured and recorded by the sensors. Saliva contains disease-related biomarkers, enabling noninvasive diagnostics. Navami et al. developed a selective Ag@HCNFs SERS substrate via coaxial electrospinning for detecting salivary nitrite [199]. The Ag@HCNFs-based SERS substrate demonstrated excellent nitrite detection capability, exhibiting a characteristic vibrational peak at 1330 cm^−1^ corresponding to the symmetric N–O stretching mode (Fig. 12g). Quantitative analysis revealed a linear concentration response (*R*^2^ = 0.9983) within clinically relevant ranges, demonstrating potential for oral cancer diagnosis (Fig. 12h). Li et al. developed a Tb-BPA-modified luminescent PAN nanofiber membrane for rapid and sensitive ATP detection in urine (Fig. 12i) [241]. The sensor showed linear luminescence quenching at 545 nm (*R*^2^ = 0.9998) (Fig. 12j), exceptional ATP specificity (Fig. 12k), and maintained stability over 7 test cycles, confirming its reliable detection capability even after multiple uses (Fig. 12l). Liu et al. developed an electrospun PA/PANI-CNTs nanofiber composite biosensor for ultrasensitive detection of Alzheimer’s biomarker Aβ42 in blood (Fig. 12m) [233]. The aptamer-functionalized sensor demonstrated a wide linear detection range (1–110 ng mL^−1^) with enhanced sensitivity through DNA-Aβ42 binding-induced conformational changes (Fig. 12n). Remarkably, the biosensor showed excellent specificity with a 16 μA response to 1 ng mL^−1^ Aβ42 versus minimal interference (< 2 μA) from 100-fold higher concentrations of other proteins (Fig. 12o).

While body fluid detection has advanced, challenges persist including analyte cross-interference, adsorption saturation instability, and sensitivity limitations. Nanofiber solutions employ Janus structures for directional fluid transport [216, 229, 243] and SERS sensors utilizing plasmonic effects for ultrasensitive multiplex detection, enabling high-performance analysis without complex preparation. Table 8 provides a comprehensive summary of the core performance parameters of electrospun nanofiber-based composite materials in body fluid signal sensing and detection in recent years.

**Table 8:** Performance evaluation of electrospun nanofiber-based composite materials on body fluid signals

Materials	Sensitivity	Working range	LOD	SNR	Other performances	Refs
PVDF + PAN/CNTs	Glucose: 7.40 μA mM^−1^ cm^−2^; Lactate: 1.86 μA mM^−1^ cm^−2^; Uric acid: 0.113 μA mM^−1^ cm^−2^	Glucose: 0–21 mM; Lactate: 5–25 mM; Uric acid: 0–20 mM	–	–	Air permeability	[243]
PVA + BTCA/AuNPs	Glucose: 47.2 μA mM^−1^	0.1–0.5 mM	0.01 mM	4.77 dB	–	[237]
PU/AuNFs	Glucose:31.94 µA (lg(mM))^−1^ cm^−2^	1–30 mM	1.01 mM	–	–	[244]
P(NIPAAm-co-NIPMAAm) + PEO + PCL/AgNCs	–	2–8 mM	2.29 mM	–	Photothermal conversion	[230]
PVDF + PVB/Ag + Au + LM	Glucose: 3.6 nA μm^−1^ Lactate: 156.6 nA mm^−1^	Glucose: 50–1000 μM Lactate: 5–25 mM pH: 3.7–8.5	–	10.7 dB	Air permeability	[235]
PMMA/Pd + Au	H_2_O_2_: 2.52 mA mM^−1^ cm^−2^ Uric Acid: 430 ± 10 μA cm^−2^ mM^−1^	H_2_O_2_: 0.01–0.3 mM Uric Acid: 0–300 μM	H_2_O_2_: 4.2 ± 0.2 μM Uric Acid: 11.6 ± 0.5 μM	–	–	[231]
PMMA/Au	pH: -28 mV pH^−1^	pH: 4–9	–	–	–	[238]

### Multi-Signal

The human body represents a highly sophisticated, multifunctional integrated system capable of simultaneously processing diverse signals to initiate appropriate responses in varying scenarios. Traditional single-modal sensors, however, fall short in providing comprehensive information for E-skin systems, limiting their functionality and practical applications. To address this limitation and achieve precise sensation, significant research efforts have been directed toward developing sensors capable of simultaneously detecting multiple stimuli. In recent years, there has been a growing emphasis on the design and fabrication of multifunctional flexible E-skin sensors that integrate multiple sensing modes. These advanced sensors can detect two or more external stimuli concurrently, enabling the acquisition of diverse and comprehensive information, which is essential for meeting the demands of real-world applications. Strategies for achieving multi-signal detection primarily involve the design of unique device architectures and the integration of multiple single-function sensors [245].

**Fig. 13:**
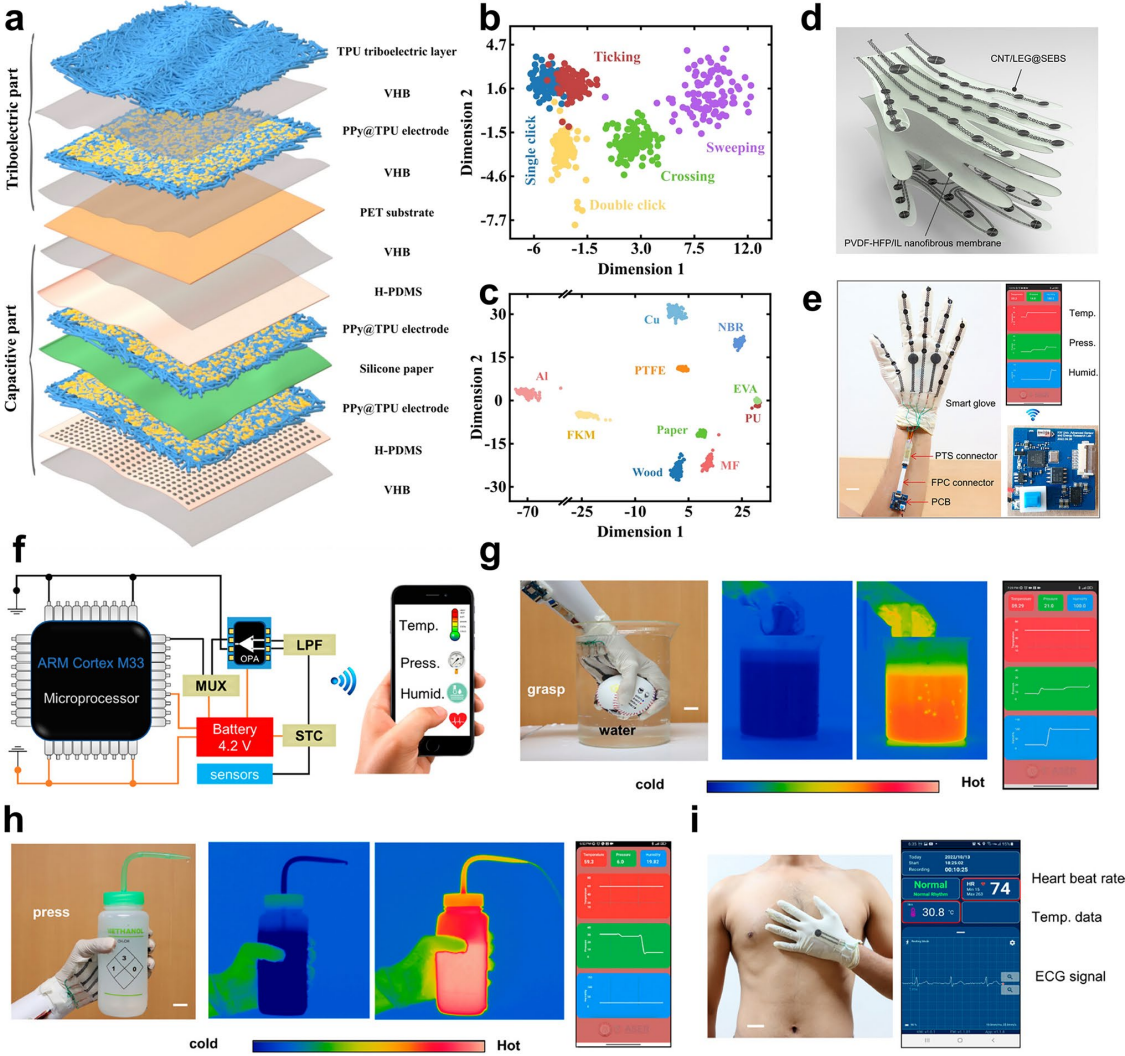
Multi-signal sensing via electrospun nanofiber-based composites sensors. **a** Illustration of the perceptron architecture. **b** Classification of different gesture recognition results using the PCA algorithm. **c** Classification of recognition results for different materials. Reproduced with permission from Ref. [246]: Copyright 2024, Donghua University. **d** Exploded view of the e-glove consisting of three stacked layers. **e** Optical photographs of the fabricated e-glove system with a custom-built PCB and connectors. **f** Wireless interface with signal processing modules. **g** Real-time ball-grasping response under thermal stimuli. **h** Bottle-squeezing detection with temperature feedback. **i** Simultaneous ECG/temperature monitoring with smartphone readout. Reproduced with permission from Ref. [247]: Copyright 2023, American Chemical Society

The design and adoption of unique device architectures enable effective decoupling of multiple stimulus signals. For instance, Ye et al. utilized electrospinning technology to fabricate and integrate a triboelectric sensor and a capacitive sensor, addressing challenges such as multimodal coordination and high-power consumption in traditional tactile sensors (Fig. 13a) [246]. The triboelectric sensor achieves noncontact gesture recognition by capturing the electron affinity of objects. By incorporating machine learning techniques, particularly principal component analysis (PCA), the sensor can distinctly differentiate various gestures (e.g., single-click, double-click, hook, swipe, and sweep) and exhibits excellent separability in a two-dimensional space. Simultaneously, the capacitive sensor detects external pressure, providing information on material properties such as hardness, softness, and deformation. Furthermore, when combined with machine learning algorithms, the sensor can effectively distinguish and analyze 10 different materials (Fig. 13b, c). Another common approach to decoupling multiple stimuli involves integrating multiple sub-sensors on a single platform. For example, Sudeep et al. developed a multimodal electronic glove sensor by combining CO_2_ laser engraving with electrospinning technology [247]. This innovative sensor is capable of detecting pressure, temperature, humidity, and ECG signals. Integrated with a Bluetooth system, it enables remote transmission of sensing data to users (Fig. 13d, e). The captured signals are processed by a microcontroller and wirelessly transmitted to the user interface via the Bluetooth module (Fig. 13f). For instance, when grasping a plastic bottle filled with hot or cold water, the pressure and temperature sensors accurately respond to grip force and temperature variations, respectively. Moreover, the e-glove successfully achieves simultaneous monitoring of human ECG and temperature signals, highlighting its significant potential for applications in intelligent prosthetics (Fig. 13g-i). The e-glove incorporates a nanofiber-based composite substrate that provides robust anchoring of sensing materials via π-π interactions, ensuring excellent interfacial stability. The device architecture features ripple-like sensing elements integrated with fully stretchable interconnects, enabling seamless deformation compliance while effectively mitigating interfacial mismatch with flexible components.

However, the challenges of decoupling multiple signals and mitigating signal cross-interference remain significant obstacles in current sensor research. At present, most soft multifunctional sensors are limited to distinguishing between two input signals, while achieving precise discrimination of three or more stimuli using a single sensing unit remains highly complex. Furthermore, coupling effects between multiple signals can lead to mutual interference, thereby compromising detection accuracy and reliability. To address these issues, the development of novel sensing mechanisms and signal compensation strategies, combined with advanced packaging technologies and machine learning algorithms, is considered a highly promising research direction. These approaches are expected to significantly enhance the multi-signal decoupling capabilities of sensors, enabling their application in complex environments. Additionally, new energy solutions are required to power fully integrated multifunctional sensor systems, with piezoelectric and triboelectric sensors offering a potential pathway forward.

## Conclusion and Outlook

To enhance traditional sensors, which are typically hard and brittle, flexible E-skin sensors have garnered significant interest from researchers. The ongoing advancements in electronic technology have elevated requirement for E-skin sensors, which now require high sensitivity, a low detection limit, a wide monitoring range, and stable signals for long-term monitoring. Additionally, these sensors are expected to possess mechanical recovery, and self-healing capabilities to withstand mechanical damage from wear and external forces. Furthermore, it is also crucial for E-skin sensors to adhere to both human skin and robotics/prosthetics firmly during the dynamic detection process, and without interfere with the normal functions of biological systems. For implantable flexible electronic sensors, in particular, biocompatible and biodegradable are essential.

**Fig. 14:**
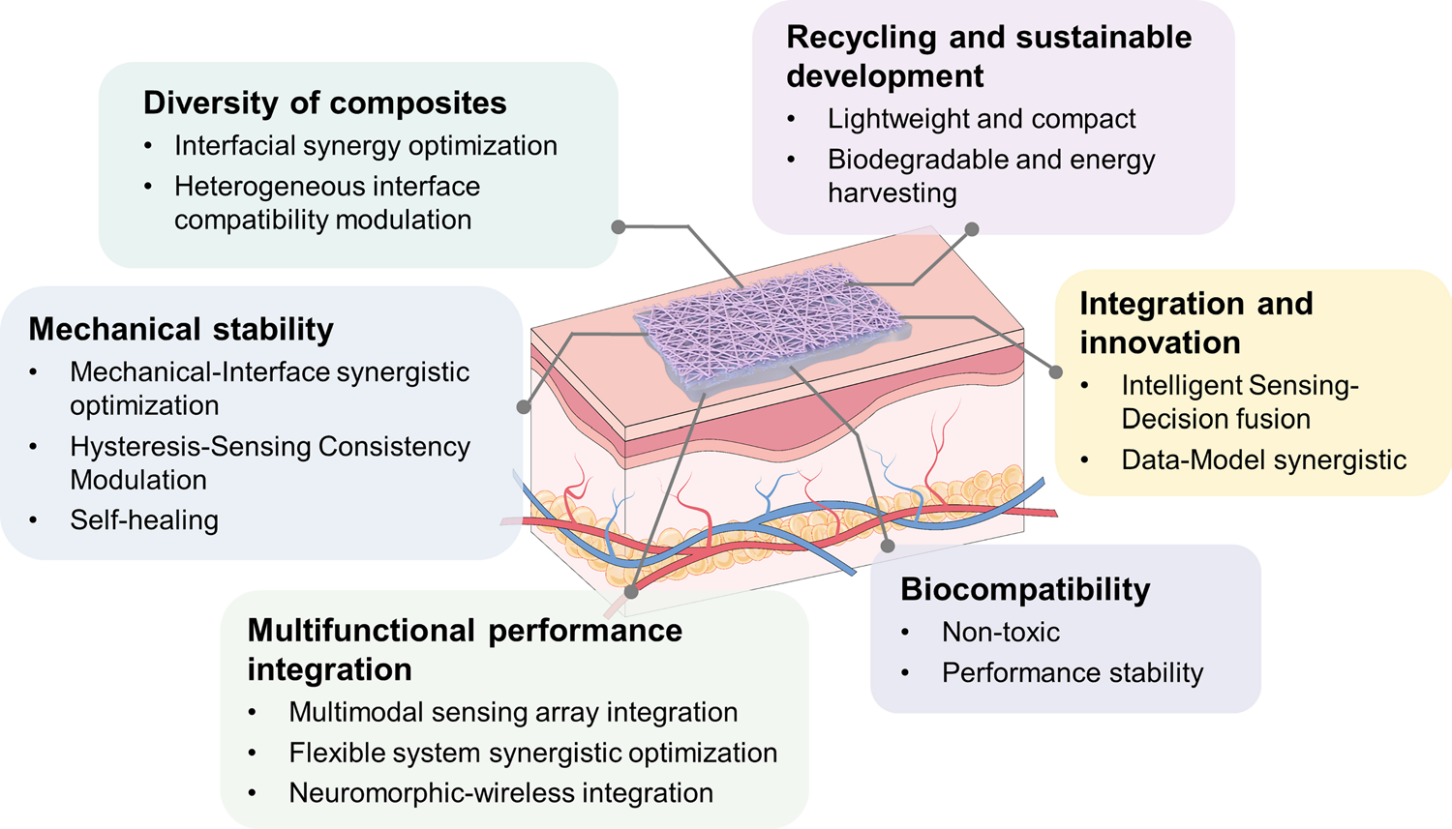
An overview of the main challenges facing nanofiber-based composite sensors

Electrospun NFMs possess large surface area, structural designability, flexibility, and the ability to be composited with other materials for performance enhancement. Thus, electrospun nanofiber-based composite materials are considered to be one of the ideal structures for E-skin sensor applications. This review first presents an overview of electrospinning including technologies, microstructure design, and fabrication of conductive nanofibers. Next, the classification of nanofiber-based composite materials, including nanofiber/hydrogel, nanofiber/aerogel, and nanofiber/metal composite materials was discussed in-depth. At last, it introduces the typical research of detecting different human signals, including physical, physiological, and body fluid signals. Despite significant progress in the development of nanofiber-based materials for flexible sensors over the past decade, several challenges remain, especially in the following aspects (Fig. 14):

### Diversity of Composites

Nanofiber-based composite sensors demonstrate enhanced performance through the synergistic combination of component advantages. Future research directions focus on three key aspects: (1) innovative structural engineering via electrospun microstructure design and surface modification, (2) the development of advanced composite architectures (e.g., nanofiber/foam and nanofiber/hydrogel foam systems), and (3) the optimization of interfacial interactions for mechanical reinforcement, signal amplification, and multifunctional integration.

However, nanofiber-based composite sensors still face significant challenges in interfacial compatibility between components. Potential solutions to address this issue may include: employing surface functionalization techniques such as plasma etching or chemical grafting, and introducing interlayers or developing novel surface modification technologies. These approaches can effectively reduce the interfacial energy barriers between heterogeneous materials, thereby enhancing interfacial bonding strength. Moreover, the advantageous properties of individual components may exhibit mutual constraints, making it challenging to achieve optimal synergistic performance. The development of intelligent responsive composite materials, combined with machine learning-assisted predictive models for material property optimization, could potentially provide a novel approach for next-generation sensor development.

### Multifunctional Performance Integration

Flexible electronics with novel functions, such as stimuli-responsive properties, self-healing abilities, mechanical toughness, and enhanced biocompatibility, are growing trend, which can extensively expand the applications of flexible sensors. However, the interference between multiple signals can affect the reliability of the signal. Therefore, there is an urgent need to devise composite materials endowed with multichannel sensing functionalities, thereby constructing flexible multimodal sensing arrays characterized by high density and spatiotemporal resolution, while ensuring effective isolation, coordination, and precise identification among the various channels. Effective isolation and coordination of multichannel signals can be achieved by developing composite materials with layered or gradient structures. This includes incorporating insulating or shielding layers to minimize signal crosstalk, integrating advanced signal processing algorithms like machine learning or deep learning for real-time analysis, and optimizing flexible circuit layouts through techniques including differential signaling or frequency modulation. Furthermore, integrating nanofiber-based composite sensors with flexible batteries, energy storage devices, and data transmission systems into a single platform, combined with low-power wireless communication technologies (e.g., Bluetooth, NFC, or 5G), could advance their applications in health monitoring. Nevertheless, challenges remain. Wireless devices are currently limited to short-distance signal transmission and are susceptible to external noise. Additionally, neuromorphic devices for processing sensor array signals are still in the proof-of-concept stage, with spiking neural network (SNN) algorithms yet to be fully developed and synaptic devices requiring further optimization. Addressing these challenges will be crucial for realizing the full potential of flexible electronics in next-generation sensing systems.

### Mechanical Stability

The ultimate goal of flexible sensors is to achieve mechanical properties similar to those of human tissue, including strain recovery, mechanical stability, and self-healing. However, there are still many challenges in practical applications. For example, the composite materials must maintain efficient signal output under repeated mechanical deformation, but prolonged stretching can lead to interfacial mismatches or cracking between components. This mismatch not only limits the fit and comfort of the device on the human body, but may also causes compression, friction or even cutting damage to the surrounding tissues due to the concentration of mechanical stress. Additionally, the performance of E-skin may be affected by the environment after long service, so keeping the flexible device sustainable and stable is also an important challenge. Although self-healing sensors are widely explored, the sensing characteristics after healing will decline inevitably, and thus should be thoroughly investigated in the future. To address these challenges, future research should focus on developing innovative strategies to enhance the performance and reliability of flexible sensors. This includes designing composite materials with a gradient modulus or adaptive interfaces to minimize stress concentrations, incorporating dynamic covalent bonds or supramolecular interactions to improve self-healing capabilities, and utilizing encapsulation techniques or environmentally stable coatings to enhance the environmental resilience and long-term sustainability of devices. Additionally, a deeper investigation into the mechanical hysteresis of electrospun nanofiber-based composites is essential to optimize their microstructure and mechanical properties, thereby unlocking new possibilities for their application in next-generation flexible sensors. However, studies on the mechanical hysteresis of electrospun nanofiber-based composite materials in sensors are still limited and require more attention. Future studies should focus on elucidating the fundamental origins of hysteresis and developing effective mitigation strategies, such as tailoring material compositions, optimizing fabrication processes, or incorporating advanced structural designs.

### Biocompatibility

Biocompatibility is a crucial consideration in the application of composite materials in the field of human signals sensing. Since these structures need to be direct or indirect contact with human tissue and may even be implanted in the body, they must be able to co-exist harmoniously with the biological environment without causing any harmful biological reactions. In addition, performance stability issues may be faced in complex human environments. For example, human skin impedance decreases after intense sweating, which may affect the reliably of the signals captured by the composite material. To address these challenges, future research should focus on enhancing the compatibility of composites with biological tissues by developing materials based on biocompatible substrates, such as biodegradable polymers and hydrogels, coupled with surface functionalization to improve their interaction with biological environments. Additionally, the design of composite materials with environmental adaptive properties, such as humidity or temperature responsiveness, along with the integration of multimodal sensing technologies, could provide robust solutions to performance stability issues by enabling signal compensation and ensuring reliable operation under varying physiological conditions.

### Recycling and Sustainable Development

Flexible composite materials sensors can be made lighter and more miniaturized, by reducing the amount of material and energy consumed in the manufacturing process. Meanwhile, the use of waste or biodegradable materials not only promotes material reuse, but also significantly reduces environmental pollution, which is crucial for the sustainable development of the flexible sensing field. Advanced manufacturing techniques, such as 3D printing and photolithography, should be a primary focus of future research to enable precise control over material usage and facilitate the development of lightweight, miniaturized sensor designs. Additionally, exploring environmentally friendly composites derived from renewable resources, such as cellulose or chitosan, and integrating them with low-power sensing technologies and energy-harvesting mechanisms, such as triboelectric or piezoelectric systems, could further reduce energy consumption and enhance the sustainability of flexible sensors.

### Integration and Innovation

The combination of machine learning and sensing technology is leading a technological revolution, and this convergence is showing great potential and value in several areas. By integrating multiple types of sensors and advanced machine learning models, systems with multi-dimensional sensing and intelligent decision-making capabilities can be built. However, the practical application of machine learning in sensing still faces challenges such as degraded model performance due to noisy sensor data and environmental variations. Enhancing data quality through multi-sensor fusion and adaptive techniques like transfer learning, alongside diversifying training data, is critical to improving robustness. Additionally, ensuring data security via encryption and privacy-preserving algorithms remains essential for sustainable integration.

In summary, with ongoing advancements in electrospinning and composite technology, the field of flexible E-skin sensors is poised to significantly impact future health monitoring and intelligent robotics.
